# Flower‐rich and diverse road verges support pollinators, but traffic speed limits the ecological benefits across Europe

**DOI:** 10.1002/eap.70279

**Published:** 2026-06-28

**Authors:** Chris Wyver, Andrijana Andrić, Carolin Biegerl, Sofia Blomqvist, Christophe Dominik, Noah Feldmann, William Fiordaliso, Mike Garratt, Andrea Holzschuh, Hanna Honchar, Reet Karise, Maja Knežević, Hanno Korten, Sarah Lescot, Egle Liiskmann, John MacArthur, Marika Mänd, Denis Michez, Erik Öckinger, Oliver Schweiger, Tea Skendžić, Henrik G. Smith, Ingolf Steffan‐Dewenter, Louise Truslove, Sanja Veselić, Dušanka Vujanović, Deepa Senapathi, Simon G. Potts

**Affiliations:** ^1^ Sustainable Land Management Department School of Agriculture, Policy and Development, University of Reading Reading UK; ^2^ Department of Biology and Ecology, Faculty of Sciences University of Novi Sad Novi Sad Serbia; ^3^ BioSense Institute, University of Novi Sad Novi Sad Serbia; ^4^ Department of Animal Ecology and Tropical Biology Biocentre, University of Würzburg Würzburg Germany; ^5^ Department of Biology Lund University Lund Sweden; ^6^ Centre for Environmental and Climate Research, Lund University Lund Sweden; ^7^ Department of Community Ecology Helmholtz Centre for Environmental Research – UFZ Halle (Saale) Germany; ^8^ German Centre for Integrative Biodiversity Research (iDiv), Halle‐Jena‐Leipzig Leipzig Germany; ^9^ Laboratory of Interaction Ecology and Global Change Research Institute for Biosciences, University of Mons Mons Belgium; ^10^ Department of Ecological Monitoring Institute for Evolutionary Ecology, NAS Ukraine Kyiv Ukraine; ^11^ Chair of Plant Health, Institute of Agricultural and Environmental Sciences, Estonian University of Life Sciences Tartu Estonia; ^12^ Laboratory of Zoology Research Institute for Biosciences, University of Mons Mons Belgium; ^13^ Department of Ecology Swedish University of Agricultural Sciences Uppsala Sweden

**Keywords:** disturbance, Europe, floral enhancement, pollinators, road verges, traffic

## Abstract

Roads are vital for human societies, yet they can also have negative impacts on the ecological communities that live in close proximity to them. Insect pollinators, which nest and forage in road verges running alongside roads, are a group of particular importance. These verges may act as an “ecological trap,” drawing insect pollinators into contact with traffic, increasing the risk of pollinator‐traffic collisions. Spanning six European regions, we evaluated the complex relationships between traffic, road verge floral composition, and surrounding land use to understand how these factors influence abundance and richness of bees, butterflies, and hoverflies sampled within road verges. Across the study, we observed 10,960 pollinators belonging to 293 species of bees, butterflies, and hoverflies. We observed greater pollinator abundance in verges with higher flower cover, and greater pollinator richness in verges with more species of flowering plants. Lower abundances of bees and butterflies and lower species richness of bees were observed when traffic speed in the adjacent road was higher. This study indicates that road verges with abundant and diverse floral resources support more abundant and diverse pollinator populations, especially on verges alongside lower speed roads. We recommend that lower speed roads should be prioritized for floral enhancements.

## INTRODUCTION

Anthropogenic activities have transformed natural ecosystems worldwide, leading to extensive habitat loss, ecological fragmentation, and widespread declines in biodiversity (Jaureguiberry et al., 2022). Human‐induced pressures such as land‐use change, intensive agriculture, and infrastructure expansion have altered landscapes at an unprecedented scale, reducing the availability of suitable habitats for many species (Goldewijk et al., 2017). These disturbances not only threaten species persistence directly, but also disrupt ecological processes such as pollination, nutrient cycling, and dispersal (Aguilar et al., 2025; Millard et al., 2021). As human societies continue to modify environments for transportation, food production, and settlement, understanding how biodiversity responds to these changes is critical for developing conservation strategies that maintain both species and ecosystem functioning.

Paved roads are a vital part of human societies, stretching for almost 40 million km globally, including over 5 million km in Europe (Central Intelligence Agency, 2024), and are responsible for a substantial proportion of the movement of people, goods, and services. The large number and linear nature of roads lead to natural habitat becoming fragmented, creating patches of land bounded on all sides by roads. Globally, over 50% of land patches created by roads are smaller than 1 km^2^ and only 7% of land patches are greater than 100 km^2^ (Ibisch et al., 2016). In the United Kingdom, it is estimated that half of the total land area is less than 216 m from a road (Phillips, Bullock, Gaston, et al., 2021).

The expansion of paved roads forces wildlife to live in close proximity to roads and their associated traffic, resulting in a range of risks, with documented issues including air quality reductions (Hooftman et al., 2018), light (Lyytimäki et al., 2012) and noise (Khan et al., 2018) pollution, habitat fragmentation, habitat loss (Ibisch et al., 2016), and wildlife‐traffic collisions (Neumann et al., 2012). Annually, as many as 350 million vertebrates in the United States (Seiler & Helldin, 2006) and 223 million birds and mammals in Europe (Grilo et al., 2020) are killed in collisions with road vehicles.

Past research has shown that insect pollinators are also impacted by these issues. For example, insect–traffic collisions may be responsible for the deaths of billions of pollinating insects every year (Baxter‐Gilbert et al., 2015), with increasing traffic intensity linked directly to increased pollinator mortality (Dániel‐Ferreira et al., 2022) and to lower abundance of pollinators within road verges (Horstmann et al., 2024; Priyadarshana et al., 2025). Aside from collision risk, one of the largest sublethal threats faced by insect pollinators in road verges is pollution (Phillips, Bullock, Osborne, & Gaston, 2021). This pollution comes in various forms including exhaust emissions, light pollution, and traffic‐induced turbulence. Exhaust emissions react with floral odors and can lead to a reduction in abundance and flower visitation of a wide range of pollinators (Ryalls et al., 2022), whereas light pollution has been shown to impair nocturnal pollinators (Straka et al., 2021). Traffic‐induced turbulence may influence pollinator movements alongside roads (Fitch & Vaidya, 2021), with pollinators finding it increasingly difficult to forage as traffic speed increases (Dargas et al., 2016; Priyadarshana et al., 2025).

For many larger species such as mammals and birds, roads often contribute towards habitat loss, whereas for smaller species such as the bees, butterflies, and hoverflies crucial for providing pollination services, road verges could provide habitat if managed appropriately (Brown et al., 2024). These areas are typically between 2 and 5 m wide and overall constitute a significant amount of land, for example between 2100 and 3000 km^2^ (approximately 1.25% of all land) in Great Britain (Phillips, Navaratnam, Hooper, et al., 2021), and around 3800 km^2^ in Germany (Wojcik & Buchmann, 2012).

Historically, many road verges have been managed with a focus on safety, aesthetics, and economics (Parr & Way, 1988) and not with biodiversity conservation in mind, with regular mowing. This often creates homogenous verges where short grass dominates with few resources for insect pollinators. An opportunity may exist to alleviate or buffer against some of the negative impacts created by roads on pollinators by shifting the focus of road verge management to be more pollinator‐friendly. A range of practices can be employed to achieve this goal, including less frequent mowing, mowing at different times of year, removing cuttings following mowing, mowing at different heights to increase structural heterogeneity, decreasing herbicide applications, and sowing wildflower mixes (Jakobsson et al., 2018; O'Sullivan et al., 2017; Priyadarshana et al., 2025).

Evidence from several studies indicates that insect pollinators respond swiftly to improvements in road verge habitat quality. Increases in local populations and associated pollination service have been seen in floristically enhanced road verges in Germany (Dietzel et al., 2023), Sweden (Horstmann et al., 2024), and the United States (Hopwood, 2008; Ries et al., 2001), particularly in urban areas where alternative resources may be scarce (Brown et al., 2024). This may also be the case in other landscapes where floral diversity is low, such as intensive agricultural landscapes lacking seminatural habitat (Phillips et al., 2019), and several studies have highlighted the potential for road verges to provide refugia for insect pollinators in intensive agricultural landscapes (Ding & Eldridge, 2022; Henriksen & Langer, 2013; Monasterolo et al., 2020; Phillips et al., 2019). It is estimated that road verges can support up to 3–4 times greater density and 1.5 times greater species richness of foraging pollinators compared with field interiors in agricultural settings (Cole et al., 2017), highlighting the potential of road verges to support both abundant and diverse pollinator communities.

This study aims to disentangle the complex relationship between insect pollinators, flowering plant diversity, nectar sugar availability, traffic density, traffic speed, and the surrounding landscape by combining systematically collected pan‐European data with remote sensing and traffic data. Specifically, we test the following hypotheses:

Hypothesis 1 Road verges with greater nectar sugar availability and higher floral diversity will support higher abundance and richness of pollinators.

Hypothesis 2 Increasing road traffic density will reduce pollinator abundance and richness in adjacent road verges.

Hypothesis 3 Higher traffic speed will reduce pollinator abundance and richness in adjacent road verges.

Hypothesis 4 Increasing proportion of cropland in the landscape will reduce pollinator abundance and richness within road verges.

## MATERIALS AND METHODS

### Site selection and characterization

**FIGURE 1:**
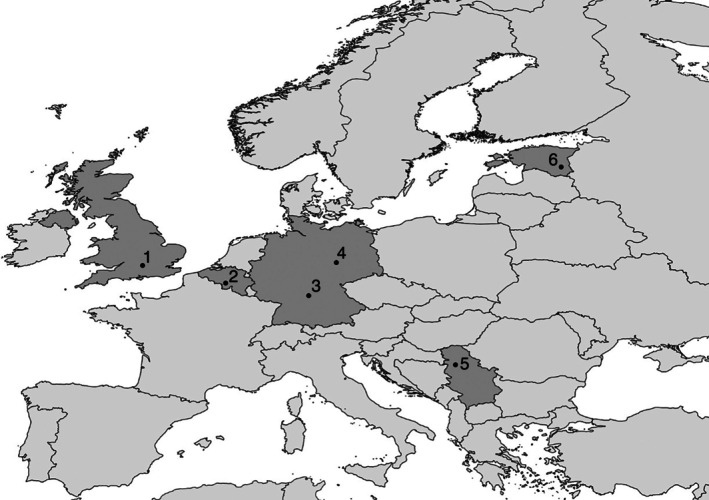
Locations of the six study regions across Europe. (1) United Kingdom, (2) Belgium, (3) Germany (Bavaria), (4) Germany (Saxony‐Anhalt), (5) Serbia, (6) Estonia (study countries in dark gray).

Six regions across five European countries, including regions in Belgium, Estonia, Serbia, the United Kingdom, and two regions in Germany were sampled, capturing a diversity in road types and land uses (Figure 1).

In each region, 24 study sites were selected to represent combinations of landscape type, road type, and verge type. Each site was surveyed three times between April and September 2023.

**FIGURE 2:**
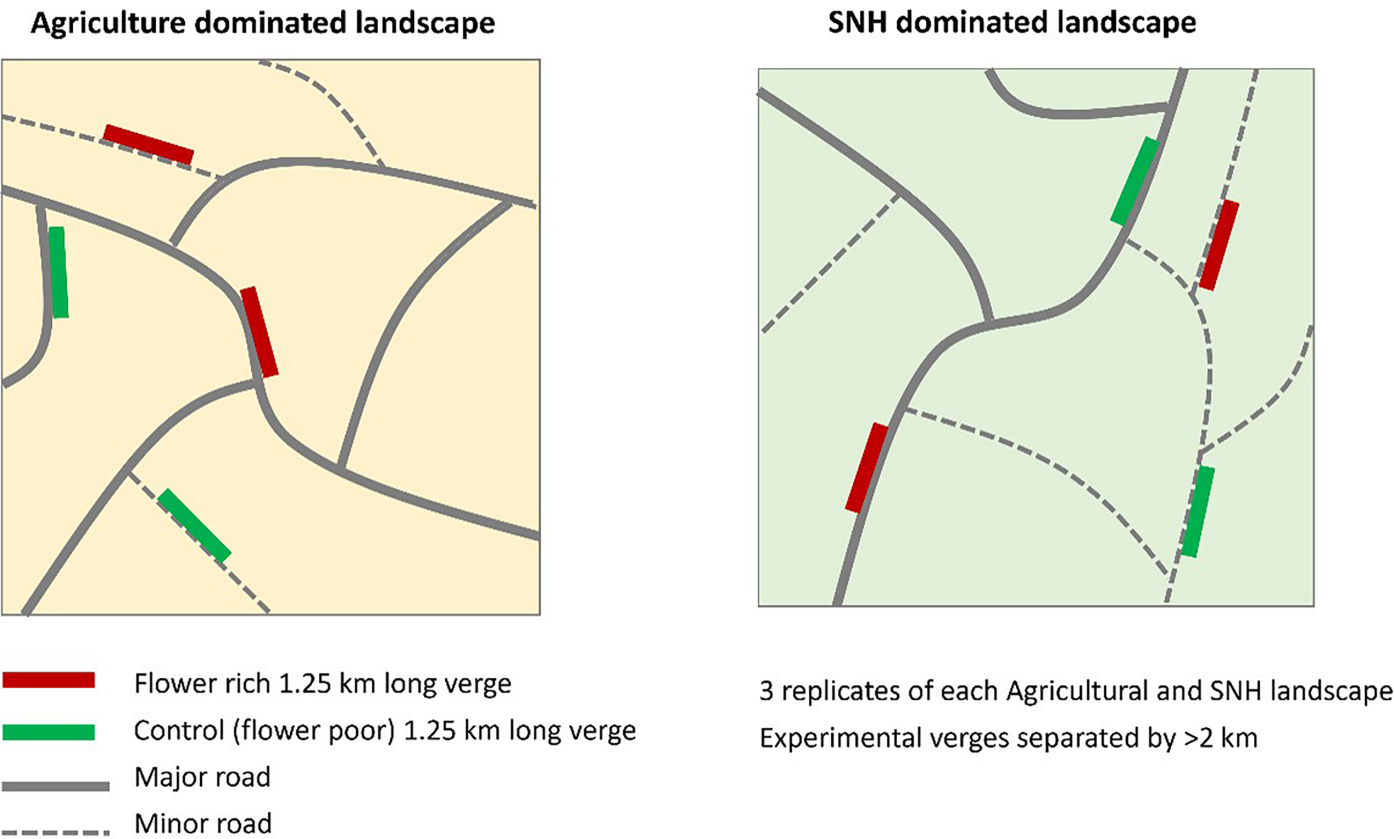
Schematic showing site selection criteria for a hypothetical landscape block. SNH, seminatural habitat.

*Landscape block*: Three separate landscape blocks were selected, each approximately 10 km^2^ in size. Within each landscape block, eight sites were selected, where possible at least 2 km from other sites, each containing a unique combination of the following characteristics:

1. *Landscape type*: Within each landscape block, two landscapes were selected containing contrasting amounts of cropland and seminatural habitat. Land use data used to quantify the amount of cropland and seminatural area were obtained from the ESRI Sentinel‐2 10‐Meter Land Use/Land Cover map for 2023 (Karra et al., 2021). The area/proportion of both cropland and seminatural within a 1‐km radius of the center of each site was calculated using QGIS (v3.40.8) to define contrasting landscapes (Appendix S1: Figure S1).
2. *Road type*: Within each landscape type, a pair of roads, each 1.25 km long, and expected to have contrasting traffic intensities were selected. Each road was single carriageway, with one lane of traffic in each direction (multilane roads were excluded on the basis of safety concerns for surveyors). At the site selection stage, roads were provisionally classified as “minor” (expected low traffic density) or “major” (expected high traffic density) based on road designation and field observations. Following site establishment, actual traffic data were extracted from the TomTom Move platform (https://move.tomtom.com), providing continuous measures of seasonal traffic density (total vehicle volume, April–September 2023) and average traffic speed (Appendix S1: Figure S2). These continuous measures were used in all subsequent analyses.
3. *Verge type*: Within each landscape type, and for each road type, two road verges with contrasting floral availability were selected. “Flower‐rich” verges consisted of verges either sown with flowering plants or managed in such a way as to promote floral resources, while “flower‐poor” verges were managed primarily for grasses and acted as controls. The categorical classification was used only for site selection. At each site, floral surveys were conducted and converted into a continuous measure of nectar sugar availability following the model developed by Baude et al. (2016). In brief, species‐specific annual nectar sugar productivity was estimated from plant traits using the model used in Baude et al. (2016) model, and combined with species' percentage cover data to calculate total nectar sugar availability per transect. This approach is described in detail in *Floral surveys and nectar sugar availability*. The continuous variable “nectar sugar availability” was then used in analyses. Figure 2 shows a hypothetical landscape block with the different combinations of landscape, road, and verge type.

### Plant–pollinator network surveys

#### Pollinator transects

A 250‐m transect was established to survey plant–pollinator networks within road verges. The exact position of the 250‐m transect within the 1.25‐km road verge varied between sites, selected as a representative section of the overall verge. A 15‐min butterfly transect was walked using a modified pollard walk technique (Pollard, 1977), recording all individuals within a 2‐m boundary of the transect, stopping the clock during handling and identification to ensure the 15 min was spent purely searching. All butterflies observed were recorded, along with the plant species they visited. Subsequently, a bee and hoverfly transect was walked, over the same 250 m, using the same method. All bees and hoverflies were recorded as well as the plant they visited. All bees, butterflies, and hoverflies were identified to species level, with individuals brought back to the laboratory for identification where necessary. Either immediately before or after the survey, traffic counts were also carried out, with all vehicles observed at one end of the 1.25‐km section of road for a 15‐min period recorded.

All surveys took place between 09:00 and 17:00 in accordance with requirements of the European Butterfly Monitoring Scheme (temperature >13°C if sunny, or >17°C if cloudy, with no precipitation in either case).

#### Floral surveys and nectar sugar availability

The flowering plant community was surveyed every 50 m along the 250 m pollinator transect using a 1 m^2^ quadrat. All plants in flower were identified, and the percentage cover of flowers of each species was estimated. From these data, the categorical variable Verge Type (“Flower” or “Grass”) was converted to a continuous variable (“nectar sugar availability”). Nectar sugar availability on the date of each transect was calculated using a model developed for British plant species by Baude et al. (2016), who collected data on annual nectar sugar productivity (in kilograms per hectare per year) for a range of plant species. A linear model was subsequently fitted as a function of plant traits to predict annual nectar sugar productivity (log_10_(*x* + 1) transformed) for species where empirical data were missing. The same plant traits used by Baude et al. (2016), namely flower shape, breeding system, life span, the degree of dicliny, the maximum height, the flowering period, and family were obtained for the plant species found in road verges from the BiolFlor database (Kühn et al., 2004).

The initial model, using the empirically collected data from Baude et al. (2016), was rerun according to the original model specifications, and traits were used to predict annual nectar sugar productivity (log_10_(*x* + 1) transformed) for species found in road verges. These values were then back transformed and multiplied by 0.27 to convert from kilograms per hectare per year to milligrams per square meter per day to represent an estimation of the nectar sugar present on the day of the survey. Where a plant species was empirically sampled by Baude et al. (2016), this value was taken over the modeled value. Using the percentage cover of each plant in each quadrat, total nectar sugar productivity was calculated, and the mean value across all five quadrats was taken as the nectar sugar availability.

### Statistical analysis

#### Factors influencing pollinator abundance and richness

All statistical analyses were carried out in R (RStudio Team, 2020, v2024.09.1.394). To test whether nectar sugar availability, surrounding land management, and traffic density and speed influence pollinator abundance in road verges, we fitted generalized linear mixed‐effects models. These models were implemented using the glmmTMB package (Brooks et al., 2017; v1.1.8). Separate models were fitted for each pollinator guild (bees, butterflies, and hoverflies) to account for potential differences in their responses to the predictors. For bees and butterflies, we used a negative binomial distribution (family = “nbinom2,” link = “log”), while for hoverflies a Poisson distribution (family = “poisson,” link = “log”) was applied.

A global model was constructed for each pollinator guild, with the total number of individuals observed per verge as the dependent variable. Fixed effects included: time of day (minutes after midnight), day of year (days since December 31st) of the pollinator survey (both modeled as linear and quadratic terms to account for diurnal and seasonal variation in pollinator activity), temperature during the survey, nectar sugar availability within the verge, proportion of surrounding cropland, daily traffic density (based on 15‐min traffic surveys to capture local variation during sampling), and seasonal traffic density and average traffic speed (both from the TomTom Move platform) for the adjacent road. All fixed effects were centered and scaled (i.e., mean subtracted and divided by SD) prior to model fitting. Random effects were structured hierarchically, with site nested within landscape, which was in turn nested within landscape block and region.

Variance inflation factors (VIFs) were calculated for the global model using the “check_collinearity” function, from the “performance” package (Lüdecke et al., 2021, v0.10.8) (Appendix S1: Table S1), for all three pollinator abundance global models; all response variables showed low correlations (VIF < 5). Conditional and marginal *R* ^2^ values for each global model were calculated using the “r2_nakagawa” function from the same package, and model checks were carried out using the “DHARMa” package (Hartig, 2022).

To test the effects of road verge management and associated traffic on pollinator diversity in road verges, a similar modeling approach was applied, with the following key differences. Species richness per verge was pooled across the season to reduce collinearity with pollinator abundance. For each guild, species richness was treated as the dependent variable. In place of nectar sugar availability, the number of unique plant species in flower at the time of the survey was used as a predictor of floral richness. We made this change based on the assumption that floral resource abundance primarily influences pollinator abundance—since greater resource availability can support larger populations (Blaauw & Isaacs, 2014)—whereas floral resource diversity is more likely to affect pollinator species richness, by offering a wider range of feeding niches (De Cauwer et al., 2006).

Daily and seasonal traffic density, average traffic speed, and the proportion of cropland surrounding the verge were also included as fixed effects. Because species richness was calculated at the seasonal level, variables related to individual survey events (e.g., time of day, date, and temperature) were excluded. Instead, latitude of the survey site was included as a fixed effect to account for latitudinal gradients in species diversity (Rohde, 1992).

Random effects were structured hierarchically, with landscape nested within landscape block nested within region. VIFs (Appendix S1: Table S2), conditional *R* ^2^, marginal *R* ^2^, and model diagnostic checks were also calculated as described previously.

## RESULTS

### Summary of pollinator communities

A total of 10,960 pollinators belonging to 293 species were recorded across the study, including 4735 bees (43.2%, 152 species), 2705 butterflies (24.6%, 65 species), and 3520 hoverflies (32.1%, 76 species). Honey bees (*Apis mellifera*) were the most frequently recorded pollinator (2444 records, 22.3%), followed by the hoverflies *Sphaerophoria scripta* (1067 records, 9.8%) and *Episyrphus balteatus* (1016 records, 9.3%). The highest number of pollinators was recorded in Serbia (3836, 35.0%), with all other locations recording between 1102 (10.1%) and 1695 (15.4%) pollinators (Appendix S1: Table S3).

### Factors influencing pollinator abundance and richness

#### Bee abundance

**FIGURE 3:**
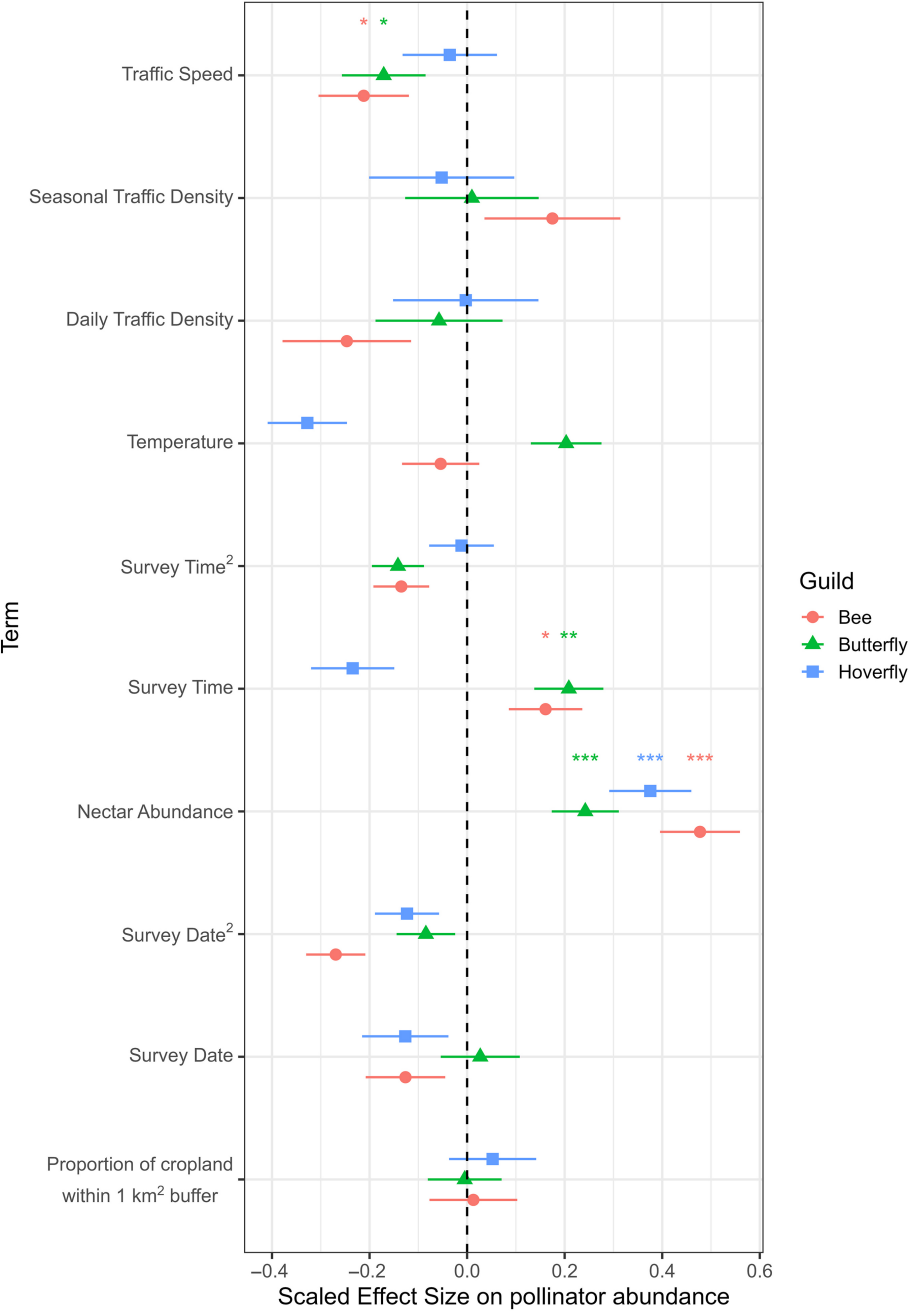
Results of generalized linear mixed‐effects models assessing the impacts of environmental predictors on pollinator abundance in road verges. Error bars represent SE. Unscaled model plots for significant environmental variables for bees can be found in Appendix S1: Figures S3 and S4, butterflies in Appendix S1: Figures S7–S9, and hoverflies in Appendix S1: Figures S11 and S12. *p ≤ 0.05; **p ≤ 0.01; ***p ≤ 0.001.

Several predictor variables had a significant effect on bee abundance within road verges (Figure 3; Appendix S1: Table S4). Nectar sugar availability had a positive impact on bee abundance (scaled model estimate = 0.48 ± 0.08, *p* < 0.001). Comparison of the scaled model coefficients showed nectar sugar availability had the largest influence on bee abundance. When nectar sugar availability is doubled from 50 to 100 mg m^−2^ day^−1^, bee abundance is predicted to increase from 16.7 ± 6.5 to 23.1 ± 9.1 individuals per transect (average across all sites when all other variables are held constant at their medians). Traffic speed also showed a significant relationship with bee abundance, with fewer individuals found in verges alongside higher speed roads (−0.21 ± 0.09, *p* = 0.022). Doubling of traffic speed from 40 to 80 km/h decreased the number of individuals seen per transect from 18.7 ± 7.9 to 10.8 ± 4.3.

Date of survey showed a significant quadratic relationship with bee abundance (−0.27 ± 0.06, *p* < 0.001). Similarly, time of survey was also significantly associated with bee abundance (Linear term: 0.16 ± 0.08, *p* = 0.032. Quadratic term: −0.14 ± 0.06, *p* = 0.018). The marginal *R* ^2^ was 0.241, while the conditional *R* ^2^ was 0.538.

#### Bee richness

**FIGURE 4:**
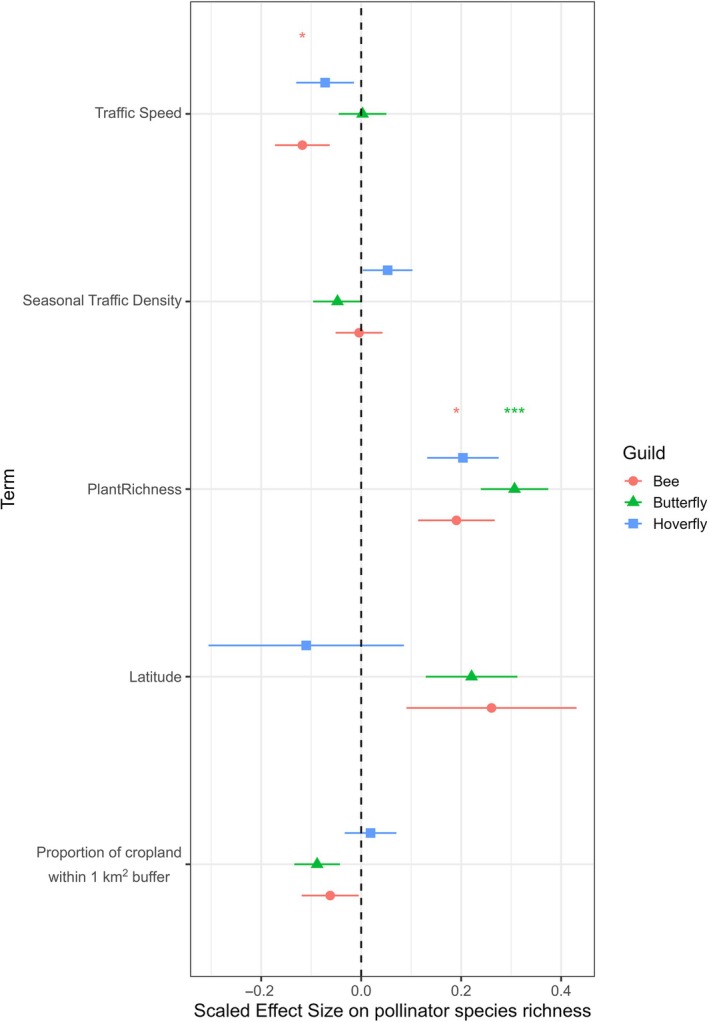
Results of linear mixed‐effects models assessing the impacts of environmental predictors on pollinator species richness in road verges. Error bars represent SE. Unscaled model plots for significant environmental variables for bees can be found in Appendix S1: Figures S5 and S6, butterflies in Appendix S1: Figure S10, and hoverflies in Appendix S1: Figure S13. *p ≤ 0.05; ***p ≤ 0.001.

The number of unique plant species in bloom across the three surveys (the proxy for flowering plant species richness) showed a positive relationship with bee species richness (0.19 ± 0.08, *p* = 0.013; Figure 4; Appendix S1: Table S5). Doubling the number of plant species in flower during a survey from 10 to 20 was predicted to increase the number of bee species found from 6.3 ± 1.4 to 7.7 ± 1.8 species. Traffic speed showed a negative relationship with bee species richness (−0.12 ± 0.06, *p* = 0.031). Doubling traffic speed from 40 to 80 km/h decreased the predicted number of bee species found from 7.9 ± 1.9 to 5.9 ± 1.3. The marginal *R* ^2^ of the global model was 0.189, while the conditional *R* ^2^ was 0.644.

#### Butterfly abundance

As with bees, butterfly abundance was positively related to nectar sugar availability (0.24 ± 0.07, *p* < 0.001; Figure 3; Appendix S1: Table S6), with a doubling of nectar sugar availability from 50 to 100 mg m^−2^ day^−1^ predicted to increase the number of butterfly individuals seen in the transect from 7.0 ± 2.6 to 8.3 ± 3.1. Traffic speed also showed a significant effect on butterfly abundance, with greater speeds related to lower butterfly abundance within the road verges (−0.17 ± 0.09, *p* = 0.046). Doubling traffic speed from 40 to 80 km/h reduced the number of butterflies observed during transects from 8.4 ± 3.3 to 5.4 ± 2.1. Survey conditions also influenced the abundance of butterflies recorded, with warmer temperatures during the pollinator survey significantly increasing butterfly abundance in road verges (0.20 ± 0.07, *p* = 0.008). An increase in temperature from 20 to 30°C was predicted to increase the average number of butterflies observed in transects from 5.2 ± 2.0 to 9.1 ± 3.5 individuals. There was a significant effect of time of day on butterfly abundance (Quadratic term: −0.14 ± 0.05, *p* = 0.007; Linear term: 0.21 ± 0.07, *p* = 0.003). The marginal *R* ^2^ was 0.158, while the conditional *R* ^2^ was 0.441.

#### Butterfly richness

Two predictors showed significant relationships with butterfly species richness (Figure 4; Appendix S1: Table S7). Firstly, butterfly species richness was significantly related to the number of unique plant species in flower across the three surveys (0.31 ± 0.07, *p* < 0.001). Doubling the number of plant species in flower during a transect from 10 to 20 is predicted to increase the number of butterfly species observed from 4.7 ± 0.8 to 6.5 ± 1.1. Secondly, the butterfly species richness was significantly related to the latitude of the survey site, with greater species richness observed as latitude increased (0.22 ± 0.09, *p* = 0.016). The marginal *R* ^2^ was 0.354 and the conditional *R* ^2^ was 0.479.

#### Hoverfly abundance

Nectar sugar availability also showed a positive impact on hoverfly abundance (0.38 ± 0.08, *p* < 0.001; Figure 3; Appendix S1: Table S8). Increasing nectar sugar availability from 50 to 100 mg m^−2^ day^−1^ is predicted to increase the number of hoverfly individuals observed from 9.1 ± 4.0 to 11.8 ± 5.2 individuals per transect. Temperature had the opposite effect on hoverfly abundance compared with butterfly abundance, with greater hoverfly abundance at lower temperatures (−0.33 ± 0.08, *p* < 0.001). Increasing temperature from 20 to 30°C was predicted to reduce the average number of hoverflies observed in a transect from 9.7 ± 4.4 to 4.0 ± 1.8 individuals. Survey time also had a significant negative linear effect, suggesting that hoverfly abundance was highest at the start of the day and declined over the course of the day (−0.23 ± 0.09, *p* = 0.006). The marginal *R* ^2^ of the global model was 0.162, and the conditional *R* ^2^ was 0.625.

#### Hoverfly richness

Hoverfly species richness increased with the number of unique plant species in flower across the three surveys (0.20 ± 0.07, *p* = 0.004; Figure 4; Appendix S1: Table S9). Increasing the number of plant species in bloom during a transect from 10 to 20 was predicted to increase the number of hoverfly species observed during a transect from 4.0 ± 1.0 to 5.0 ± 1.3. The marginal *R* ^2^ was 0.140, while the conditional *R* ^2^ was 0.600.

## DISCUSSION

Bringing together data collected across six European regions, this study provided mixed support for the hypotheses, with support for Hypothesis 1: *Road verges with greater nectar sugar availability and higher floral diversity will support higher abundance and richness of pollinators*, and Hypothesis 3: *Higher traffic speed will reduce pollinator abundance and richness in adjacent road verges*. Our results provided no support for Hypothesis 2: *Increasing road traffic density will reduce pollinator abundance and richness in adjacent road verges* or Hypothesis 4: *Increasing proportion of cropland in the landscape will reduce pollinator abundance and richness within road verges*. The findings relating to each hypothesis are discussed in more detail in the subsequent sections.

### Floral resource abundance and diversity promotes abundant and diverse pollinator communities

This study shows that increased nectar sugar availability and floral diversity within road verges positively influences pollinator abundance and species richness. This aligns with widely reported findings linking abundant and diverse floral resources to abundant and diverse populations of bees, butterflies, and hoverflies (Rollin et al., 2019; Wu et al., 2021). In addition, our results strengthen the argument that road verges managed to provide floral resources can support pollinators, as shown in previous studies (Horstmann et al., 2024, 2025; Priyadarshana et al., 2025). Wider landscape composition had no effect on abundance or diversity of any guild, indicating that the availability and diversity of floral resources within the verge itself are the primary drivers of pollinator communities within the verge, regardless of the surrounding landscape context, providing support for Hypothesis 1 (*Road verges with greater nectar sugar availability and higher floral diversity will support higher abundance and richness of pollinators*).

### Mixed impacts of traffic on pollinators within road verges

That there was no evidence for an effect of traffic density (at the spatial scale examined) on the pollinator community within road verges (either during the survey, or across the pollinator flight season) was contrary to Hypothesis 2 (*Increasing road traffic density will reduce pollinator abundance and richness in adjacent road verges*) and previous studies assessing the impact of traffic on pollinators and other insects (Horstmann et al., 2024; Martin et al., 2018). One possible explanation is that pollinators in these habitats may tolerate constant moderate disturbance from traffic, including turbulence, noise, and vibration, and can maintain normal foraging and flight behavior under these conditions, and that they could tolerate conditions within the range of conditions observed in this study. This is supported by previous research, which found no impact of several pollutants common in road verges on pollinator activity, including noise and dust (Phillips, Bullock, Osborne, & Gaston, 2021).

Although pollinators may be able to tolerate some disturbances in road verges such as noise and dust, traffic speed had a negative impact on bee and butterfly abundance and bee species richness (in support of Hypothesis 3—*Higher traffic speed will reduce pollinator abundance and richness in adjacent road verges*), indicating that, at higher levels of disturbance caused by fast‐moving traffic, some species may experience reduced activity in road verges. Our results corroborate previous research, which found decreased flower visit duration by pollinators under simulated turbulence (Phillips, Bullock, Osborne, & Gaston, 2021), and reduced pollination of lark daisy (*Centratherum punctatum*) in Amazonian road verges when traffic speed increases (Dargas et al., 2016).

By potentially avoiding road verges, or because insects are less active in verges alongside high‐speed roads, pollinators may go undetected during surveys. It is possible that the turbulence created by high‐speed traffic limits the ability of pollinators to forage. Previous research has shown that wind speed reduces the time honey bees spend in flight as flower handling time increases with greater turbulence (Hennessy et al., 2021), and it is likely that the turbulence created by fast‐moving vehicles creates conditions comparable to the higher wind treatments, and vice versa for slower moving vehicles in the experiment by Hennessy et al. (2021).

Interestingly, hoverfly abundance did not appear to be significantly impacted by traffic speed. Research into the aerodynamics of hoverflies shows individuals are capable of a fast stabilization response (Zhang et al., 2023), meaning they may be less impacted by gusts of wind created by fast‐moving traffic compared with bees and butterflies.

Increased turbulence at higher speeds may not be the only factor linked to the reduced bee and butterfly activity in road verges seen in this study. Increased traffic speed has also been linked to increased vibration (Rivas et al., 2017) and noise (traffic speed has incorporated as a factor in models predicting traffic noise; Mann & Singh, 2022), which may impact pollinator behavior in roads, though the evidence to support this theory is inconclusive (Davis et al., 2018; Phillips, Bullock, Gaston, et al., 2021).

### No impact of surrounding land use on pollinator abundance or richness

Our study found no significant relationship between the proportion of cropland and the abundance or richness of any pollinator guild (at the spatial scale and using the land‐use metrics applied here), contrary to the expectation of Hypothesis 4 (*Increasing proportion of cropland in the landscape will reduce pollinator abundance and richness within road verges*). This may partly reflect the land‐use data used, as the ESRI Sentinel‐2 10‐Meter Land Use/Land Cover map “cropland” category encompasses a wide range of agricultural habitats that likely differ in floral and nesting resource availability, ranging from cereal to mass‐flowering crops. Due to the limited availability of fine‐scale, annual, pan‐European land‐use datasets, the ESRI Sentinel‐2 10‐Meter Land Use/Land Cover map product was used to provide year appropriate, high‐resolution coverage across the study region.

These results contrast with previous studies, which found that landscape‐level greenness positively influenced butterfly abundance (Priyadarshana et al., 2025). Furthermore, they found that landscape‐level effects are often scale‐dependent, showing significant impacts at 500–1000‐m radii but not at smaller scales (50–250 m). Future work should investigate scale‐dependent responses of pollinators to broader landscape composition, though this goes beyond the scope of the current study.

### Management recommendations

In light of the findings of this study, and in combination with previously published work, we propose several context‐dependent recommendations for the management of roads and roadside habitats that may provide benefits to pollinators and wider insect diversity, particularly at the spatial scale of individual road verges examined here. Given the positive associations between nectar sugar availability and pollinator abundance, and between flowering plant diversity and pollinator diversity, management of road verges to provide abundant and diverse floral resources throughout the pollinator flight season should be encouraged where driver safety permits. Although this has already been highlighted (Horstmann et al., 2024, 2025; Phillips et al., 2019; Priyadarshana et al., 2025), the present study contributes to the growing body of evidence that pollinator‐friendly road verge management can deliver meaningful ecological benefits.

Reducing traffic speed could further promote pollinator activity along road verges based on the conditions experienced in this study, although implementing such measures on a large scale is unlikely. Given the limited resources available for verge management and the large extent of land involved, a more focused strategy may be to implement interventions on the verges adjoining roads where traffic speeds are already low, maximizing the benefits of road verges with the most optimal traffic conditions for pollinators. Alternatively, promoting verges with varied vegetation height may be an option to buffer the disturbance caused by high‐speed traffic (Priyadarshana et al., 2025).

Such management could take several forms. Altered mowing regimes, as suggested by Horstmann et al. (2024) and Priyadarshana et al. (2025), may extend flowering opportunities and increase nectar sugar provision throughout the year. Increasing verge width has also been shown to support higher plant species richness and pollinator abundance (Monasterolo et al., 2020), whilst also providing habitat for wider insect populations (Saarinen et al., 2005). Furthermore, evidence suggests that bees and butterflies respond rapidly to improvements in verge quality (Brown et al., 2024), indicating that targeted management could yield measurable benefits within a relatively short timeframe.

It is also important that interventions are tailored to local ecological contexts. Bespoke management that considers the composition of plant and pollinator communities has been shown to increase the likelihood of success (Bowgen et al., 2022). Key considerations include phenology (ensuring floral resources coincide with pollinator activity), species composition (providing appropriate host plants and prioritizing native flora when sowing), and driver safety (maintaining visibility at junctions and along sightlines). In conclusion, this study presents evidence that well managed road verges can support abundant and diverse pollinator populations at local scales, although outcomes are likely to vary with traffic conditions, landscape context, and management feasibility. Further research is recommended to assess the risk of collisions with traffic to bees, butterflies, and hoverflies and to examine the impacts of landscape at different spatial scales than tested here. Therefore, identifying potential high‐value verges and implementing bespoke management is recommended to safeguard pollinators and the vital services they provide.

## CONFLICT OF INTEREST STATEMENT

The authors declare no conflicts of interest.

## Supporting information


Appendix S1.

## Data Availability

Data and code (Wyver et al., 2025) are available in Zenodo at https://doi.org/10.5281/zenodo.17521621.
