# Supplementary material for: Flower‐rich and diverse road verges support pollinators, but traffic speed limits the ecological benefits across Europe
Source: Ecol Appl. 2026 Jun 28;36(4):e70279. doi: 10.1002/eap.70279 (PMC13310488; doi:10.1002/eap.70279)
Supplement: Supplementary file 1 — Appendix S1. [file EAP-36-e70279-s001.pdf]

Appendix S1

Flower-rich and diverse road verges support pollinators, but traffic speed limits the ecological benefits across Europe

Chris Wyver, Andrijana Andrić, Carolin Biegerl, Sofia Blomqvist, Christophe Dominik, Noah Feldmann, William Fiordaliso, Mike Garratt, Andrea Holzschuh, Hanna Honchar, Reet Karise, Maja Knežević, Hanno Korten, Sarah Lescot, Egle Liiskmann, John MacArthur, Marika Mänd, Denis Michez, Erik Öckinger, Oliver Schweiger, Tea Skendžić, Henrik G. Smith, Ingolf Steffan-Dewenter, Louise Truslov[1], Sanja Veselić, Dušanka Vujanović, Deepa Senapathi, Simon G. Potts

Ecological Applications

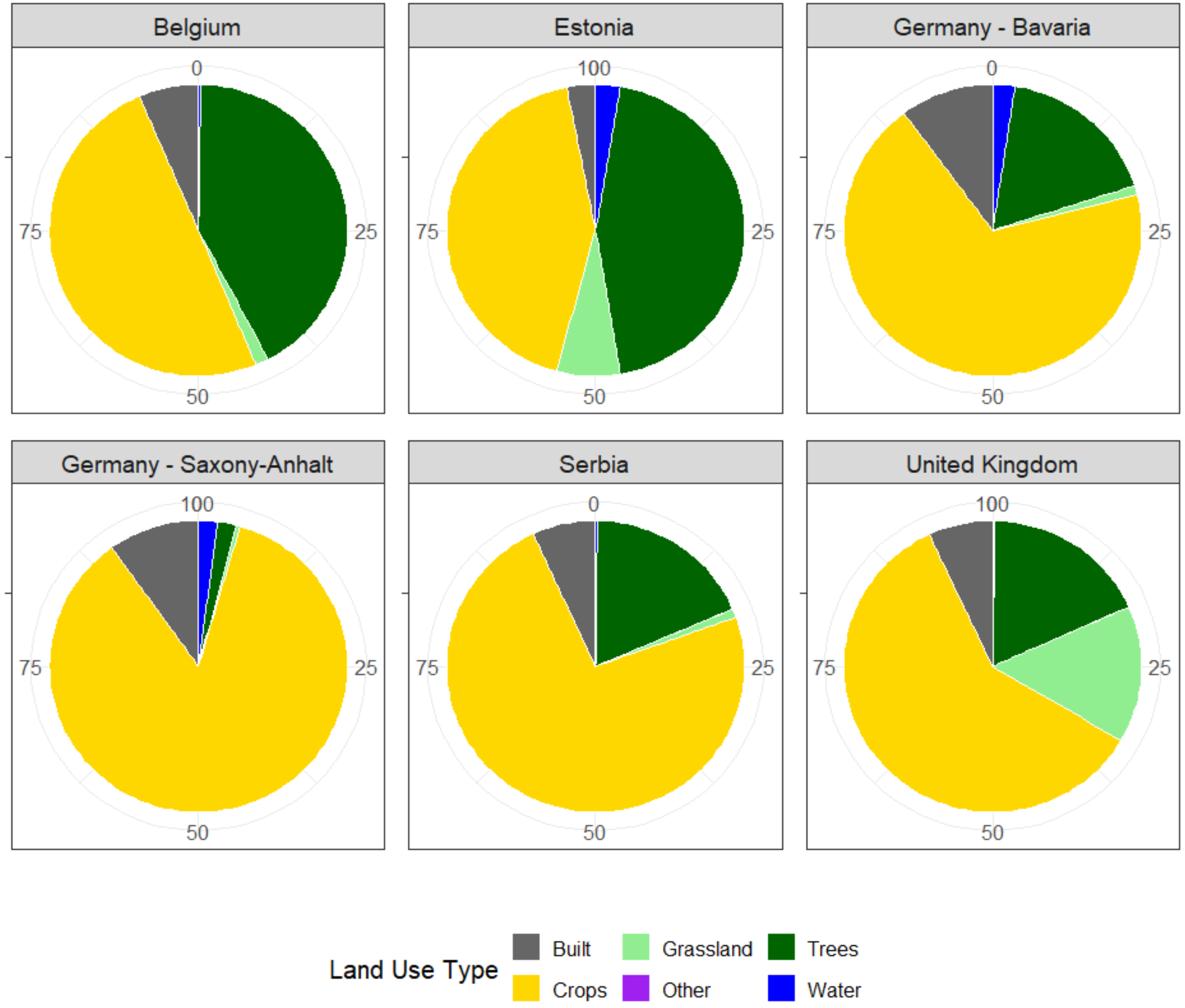

Figure S1. Mean proportion of land use types within 1 km^2 buffer surrounding each verge - grouped by region. Derived from ESRI Sentinel-2 10-Meter Land Use/Land Cover map for 2023 (Karra et al., 2021).

Karra, K., C. Kontgis, Z. Statman-Weil, J. C. Mazzariello, M. Mathis, & S. P. Brumby. 2021. Global land use/land cover with Sentinel 2 and deep learning. *2021 IEEE International Geoscience and Remote Sensing Symposium IGARSS*, 4704–4707.

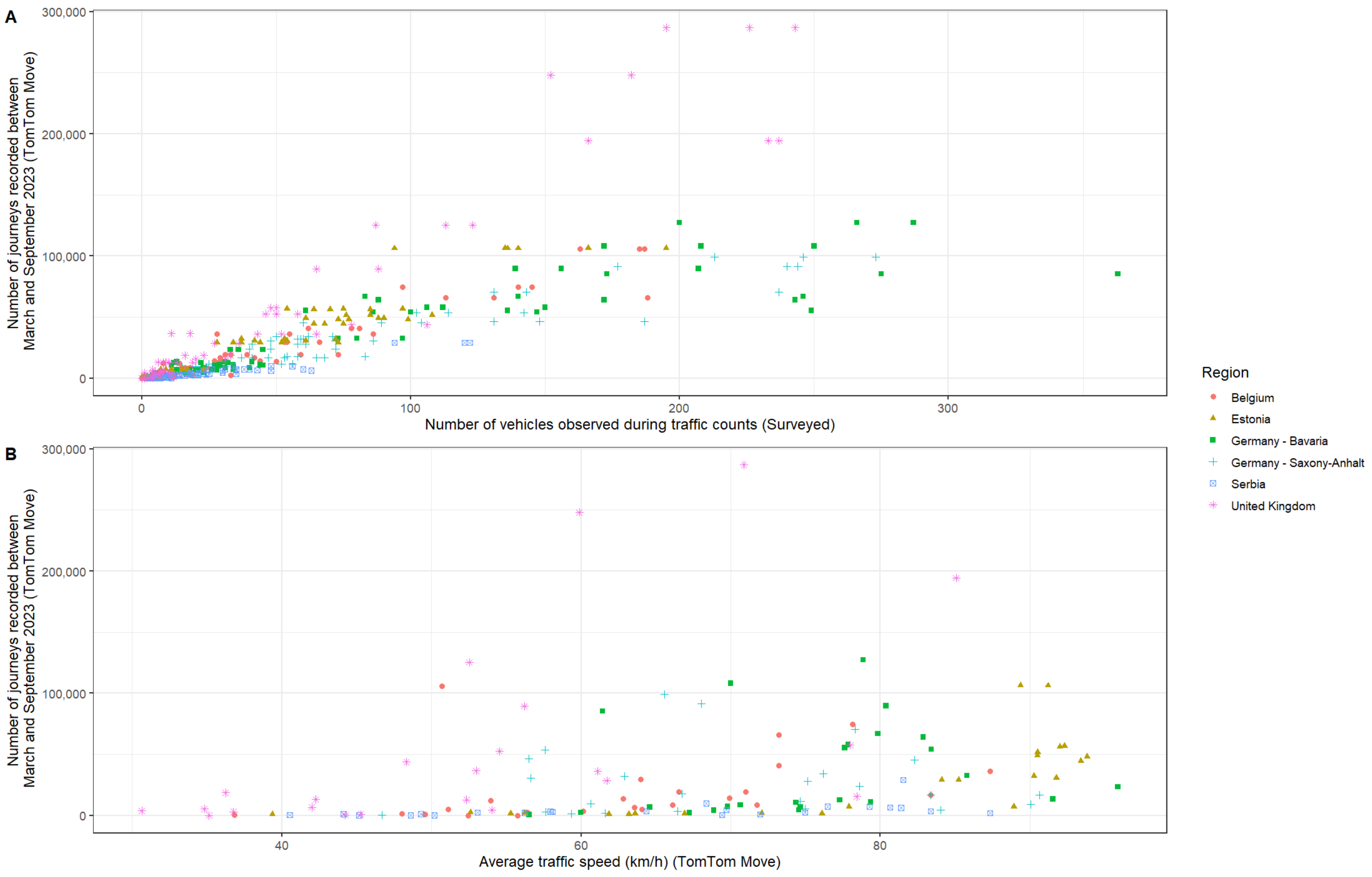

Figure S2. A) Relationship between traffic density obtained from the TomTom Move platform and surveyed traffic density. B) Relationship between traffic density and average traffic speed, both obtained from the TomTom Move platform

Table S1. Variance Inflation Factors of explanatory variables for models used to estimate effects of road verge, traffic and environment on pollinator abundance within road verges. Figures in brackets indicate 95% confidence intervals

Guild	Nectar Abundance	Seasonal Traffic Density	Traffic Speed	Daily Traffic Density	Proportion of Cropland	Survey Date	Survey Date²	Temperature	Survey Time	Survey Time²
**Bee**	1.05 [1.01, 1.39]	3.71 [3.17, 4.38]	1.37 [1.24, 1.58]	3.49 [2.99, 4.12]	1.20 [1.10, 1.38]	1.13 [1.05, 1.32]	1.12 [1.05, 1.32]	1.34 [1.21, 1.54]	1.33 [1.21, 1.53]	1.14 [1.06, 1.33]
**Butterfly**	1.08 [1.02, 1.31]	4.13 [3.52, 4.88]	1.43 [1.29, 1.65]	3.86 [3.30, 4.56]	1.16 [1.08, 1.35]	1.10 [1.04, 1.31]	1.17 [1.08, 1.35]	1.25 [1.14, 1.43]	1.38 [1.25, 1.59]	1.25 [1.14, 1.43]
**Hoverfly**	1.04 [1.00, 1.44]	3.71 [3.18, 4.39]	1.37 [1.24, 1.58]	3.64 [3.12, 4.30]	1.22 [1.12, 1.40]	1.09 [1.03, 1.31]	1.14 [1.06, 1.33]	1.33 [1.20, 1.52]	1.28 [1.17, 1.48]	1.13 [1.05, 1.32]

Table S2. Variance Inflation Factors of explanatory variables for models used to estimate effects of road verge, traffic and environment on pollinator richness within road verges. Numbers in brackets indicate 95% confidence intervals

Guild	Plant Species Richness	Seasonal Traffic Density	Traffic Speed	Proportion of Cropland	Latitude
**Bee**	1.04 [1.00, 3.30]	1.26 [1.11, 1.64]	1.31 [1.14, 1.69]	1.16 [1.04, 1.57]	1.04 [1.00, 3.92]
**Butterfly**	1.15 [1.04, 1.57]	1.21 [1.07, 1.59]	1.33 [1.15, 1.72]	1.14 [1.03, 1.57]	1.13 [1.03, 1.58]
**Hoverfly**	1.15 [1.04, 1.58]	1.02 [1.00, 22.87]	1.16 [1.04, 1.58]	1.24 [1.09, 1.63]	1.48 [1.25, 1.90]

Table S3. Counts of each species caught in pollinator transects, split by region.

Species	Group	Belgium	Germany - Saxony-Anhalt	UK	Germany - Bavaria	Estonia	Serbia	Total
Aglais io	Butterfly	11	1	6	0	0	0	18
Aglais urticae	Butterfly	0	0	2	1	25	0	28
Andrena alfkenella	Bee	0	0	14	2	0	0	16
Andrena bicolor	Bee	1	0	4	0	2	0	7
Andrena bicornis	Bee	0	0	0	1	0	0	1
Andrena cineraria	Bee	0	0	0	11	0	0	11
Andrena decipiens	Bee	0	0	0	1	0	0	1
Andrena denticulata	Bee	0	0	0	0	4	0	4
Andrena dorsalis	Bee	0	0	0	0	0	1	1
Andrena dorsata	Bee	4	7	11	1	0	0	23
Andrena flavipes	Bee	0	61	2	7	0	3	73
Andrena floricola	Bee	0	33	0	0	0	0	33
Andrena hattorfiana	Bee	0	0	0	0	11	2	13
Andrena hypopolia	Bee	0	7	0	0	0	0	7
Andrena labiata	Bee	0	0	0	2	0	0	2
Andrena lagopus	Bee	0	0	0	0	0	1	1
Andrena minulata	Bee	1	0	0	0	0	0	1
Andrena minutula	Bee	0	29	0	4	4	0	37
Andrena minutuloides	Bee	0	14	0	2	1	0	17
Andrena nitida	Bee	0	0	0	0	0	1	1
Andrena nitidiuscula	Bee	0	0	0	10	0	0	10
Andrena niveata	Bee	0	5	0	0	0	0	5
Andrena polita	Bee	0	0	0	1	0	0	1
Andrena ruficrus	Bee	0	0	0	0	1	0	1
Andrena scotica	Bee	0	0	1	0	0	0	1
Andrena semilaevis	Bee	0	0	7	0	1	0	8
Andrena subopaca	Bee	0	0	0	2	0	0	2
Andrena taraxaci	Bee	0	0	0	0	0	2	2
Andrena viridescens	Bee	0	0	0	1	0	0	1
Andrena wilkella	Bee	0	0	0	1	4	0	5
Anthocharis cardamines	Butterfly	0	0	0	0	0	2	2
Anthophora aestivalis	Bee	0	0	0	2	0	0	2
Anthophora plumipes	Bee	0	0	0	0	0	2	2
Anthophora salviae	Bee	0	0	0	0	0	2	2
Apatura ilia	Butterfly	0	0	0	0	1	0	1
Aphantopus hyperantus	Butterfly	2	0	3	0	90	1	96
Apis mellifera	Bee	231	141	122	194	158	1598	2444
Aporia crataegi	Butterfly	0	0	0	0	6	1	7
Araschnia levana	Butterfly	8	0	0	0	0	0	8
Argynnis adippe	Butterfly	0	0	0	0	1	0	1
Argynnis aglaja	Butterfly	0	0	0	0	4	0	4
Argynnis paphia	Butterfly	0	0	1	2	10	0	13
Aricia agestis	Butterfly	0	0	11	1	0	1	13
Baccha elongata	Hoverfly	2	0	0	0	0	0	2
Biastes brevicornis	Bee	0	0	0	0	0	3	3
Boloria dia	Butterfly	0	0	0	0	0	6	6
Bombus agrorum	Bee	0	0	0	0	0	1	1
Bombus barbutellus	Bee	0	0	0	0	1	0	1
Bombus bohemicus	Bee	0	1	0	0	14	0	15

Bombus campestris	Bee	0	0	0	0	12	0	12
Bombus confusus	Bee	0	0	0	0	3	0	3
Bombus haematurus	Bee	0	0	0	0	0	5	5
Bombus hortorum	Bee	3	0	7	7	4	0	21
Bombus humilis	Bee	0	0	0	1	1	0	2
Bombus hypnorum	Bee	1	0	3	0	3	0	7
Bombus jonellus	Bee	0	0	0	0	5	0	5
Bombus lapidarius	Bee	30	107	22	30	27	5	221
Bombus lucorum	Bee	0	2	0	0	18	0	20
Bombus muscorum	Bee	0	0	0	0	1	0	1
Bombus norvegicus	Bee	0	0	0	0	1	0	1
Bombus pascuorum	Bee	74	11	104	25	34	30	278
Bombus pratorum	Bee	3	0	20	3	4	1	31
Bombus quadricolor	Bee	0	0	0	0	1	0	1
Bombus ruderarius	Bee	0	0	0	0	10	0	10
Bombus rupestris	Bee	0	0	3	0	3	0	6
Bombus sensus stricto	Bee	38	0	0	0	0	0	38
Bombus soroeensis	Bee	0	15	0	0	13	0	28
Bombus subterraneus	Bee	0	0	0	0	1	0	1
Bombus sylvarum	Bee	0	3	0	110	11	0	124
Bombus sylvestris	Bee	2	0	0	0	0	0	2
Bombus terrestris	Bee	0	73	140	59	8	67	347
Bombus vestalis	Bee	6	0	11	2	2	0	21
Bombus veteranus	Bee	0	0	0	0	3	0	3
Brenthis daphne	Butterfly	0	0	0	0	0	3	3
Brenthis ino	Butterfly	4	0	0	0	5	0	9
Carcharodus alceae	Butterfly	0	0	0	1	0	0	1
Celastrina argiolus	Butterfly	18	0	6	0	0	15	39
Ceratina cyanea	Bee	0	0	0	1	0	6	7
Ceratina nigrolabiata	Bee	0	0	0	0	0	4	4
Cheilosia albitarsis	Hoverfly	0	0	0	0	0	4	4
Cheilosia illustrata	Hoverfly	5	0	0	0	0	0	5
Cheilosia paga	Hoverfly	3	0	1	1	1	0	6
Cheilosia proxima	Hoverfly	3	0	0	0	0	0	3
Cheilosia soror	Hoverfly	0	0	1	0	0	0	1
Cheilosia verlis	Hoverfly	0	0	0	0	5	0	5
Cheilosia vulpina	Hoverfly	0	0	2	0	0	0	2
Chelostoma campanularum	Bee	0	0	0	0	1	0	1
Chelostoma florisomne	Bee	0	0	0	0	0	6	6
Chrysogaster solstitialis	Hoverfly	7	0	3	0	0	0	10
Chrysotoxum bicinctum	Hoverfly	0	0	4	0	0	0	4
Chrysotoxum cautum	Hoverfly	0	0	0	1	0	2	3
Chrysotoxum elegans	Hoverfly	0	0	0	0	2	0	2
Chrysotoxum festivum	Hoverfly	0	0	0	2	3	1	6
Chrysotoxum gracile	Hoverfly	0	0	0	1	0	0	1
Chrysotoxum tricinctum	Hoverfly	0	0	0	0	1	0	1
Chrysotoxum vernale	Hoverfly	0	0	0	0	0	1	1
Chrysotoxum verralli	Hoverfly	0	0	0	1	0	0	1
Coelioxys inermis	Bee	0	0	0	0	1	0	1
Coenonympha arcania	Butterfly	0	0	0	0	2	0	2
Coenonympha glycerion	Butterfly	0	0	0	0	15	0	15
Coenonympha pamphilus	Butterfly	4	32	1	37	30	67	171
Colias croceus	Butterfly	0	0	0	7	0	20	27

Colias hyale	Butterfly	0	0	0	0	4	2	6
Colletes daviesanus	Bee	1	0	0	1	0	0	2
Cupido argiades	Butterfly	0	0	0	0	2	3	5
Cupido minimus	Butterfly	0	0	1	0	1	0	2
Dasypoda hirtipes	Bee	0	11	0	2	1	0	14
Dasysyrphus albostriatus	Hoverfly	0	0	0	1	0	0	1
Dasysyrphus tricinctus	Hoverfly	0	0	0	0	4	0	4
Epeoloides coecutiens	Bee	0	0	0	0	1	0	1
Epistrophe grossularie	Hoverfly	0	0	0	0	1	0	1
Epistrophe nitidicollis	Hoverfly	0	0	0	0	1	0	1
Episyrphus balteatus	Hoverfly	230	89	376	69	28	224	1016
Eristalinus aeneus	Hoverfly	0	0	0	0	2	1	3
Eristalinus sepulchralis	Hoverfly	0	0	0	2	0	0	2
Eristalis anthophorina	Hoverfly	0	0	0	0	1	0	1
Eristalis arbustorum	Hoverfly	8	0	1	1	3	207	220
Eristalis horticola	Hoverfly	0	0	1	0	0	0	1
Eristalis nemorum	Hoverfly	12	0	0	0	0	0	12
Eristalis pertinax	Hoverfly	117	0	11	0	1	0	129
Eristalis pertinax/tenax	Hoverfly	0	0	1	0	0	0	1
Eristalis tenax	Hoverfly	31	3	8	27	1	290	360
Eucera chrysopyga	Bee	0	0	0	0	0	1	1
Eucera longicornis	Bee	0	0	0	0	5	0	5
Eucera nigrescens	Bee	0	0	0	0	0	10	10
Eumerus strigatus	Hoverfly	0	0	0	1	0	0	1
Eupeodes corollae	Hoverfly	15	0	6	16	0	95	132
Eupeodes luniger	Hoverfly	6	0	3	0	0	13	22
Favonius quercus	Butterfly	0	0	0	0	0	1	1
Gonepteryx rhamni	Butterfly	43	0	3	0	37	3	86
Halictus leucaheneues	Bee	0	1	0	0	0	0	1
Halictus leucozonium	Bee	0	0	0	1	0	0	1
Halictus maculatus	Bee	0	0	0	1	4	0	5
Halictus quadricinctus	Bee	0	0	0	3	2	5	10
Halictus rubicundus	Bee	1	0	0	0	4	0	5
Halictus scabiosae	Bee	0	28	0	16	0	0	44
Halictus sexcinctus	Bee	0	7	0	10	0	0	17
Halictus simplex	Bee	0	18	0	6	0	0	24
Halictus subauratus	Bee	0	6	0	14	0	0	20
Halictus tumulorum	Bee	0	2	1	9	16	0	28
Helophilus pendulus	Hoverfly	0	0	0	1	18	0	19
Helophilus trivittatus	Hoverfly	0	0	0	1	0	19	20
Heriades truncorum	Bee	0	2	0	4	2	1	9
Heteropterus morpheus	Butterfly	0	0	0	0	3	0	3
Hoplitis adunca	Bee	0	0	0	1	0	0	1
Hylaeus brevicornis	Bee	0	1	0	0	0	1	2
Hylaeus communis	Bee	1	4	1	0	3	0	9
Hylaeus cornutus	Bee	0	0	0	1	0	0	1
Hylaeus dilatatus	Bee	0	0	0	1	0	0	1
Hylaeus duckei	Bee	0	0	0	2	0	0	2
Hylaeus gredleri	Bee	0	0	0	1	0	0	1
Hylaeus nigritus	Bee	0	0	0	0	1	0	1
Hylaeus pictipes	Bee	0	0	0	0	1	0	1
Hylaeus variegatus	Bee	0	1	0	7	0	0	8
Iphiclides podalirius	Butterfly	1	0	0	0	0	13	14

Issoria lathonia	Butterfly	0	2	0	1	2	7	12
Lasioglossum albipes	Bee	0	0	4	0	10	0	14
Lasioglossum calceatum	Bee	16	2	2	7	4	0	31
Lasioglossum costulatum	Bee	0	0	0	1	0	0	1
Lasioglossum fratellum	Bee	0	0	0	0	9	0	9
Lasioglossum fulvicorne	Bee	1	0	2	0	1	0	4
Lasioglossum glabriusculum	Bee	0	0	0	6	0	0	6
Lasioglossum interruptum	Bee	0	3	0	1	0	0	4
Lasioglossum laticeps	Bee	0	5	0	1	2	0	8
Lasioglossum lativentre	Bee	1	0	1	2	0	0	4
Lasioglossum leucopus	Bee	0	0	1	0	11	0	12
Lasioglossum leucozonium	Bee	0	1	3	4	2	0	10
Lasioglossum lineare	Bee	0	2	0	0	0	0	2
Lasioglossum malachurum	Bee	0	48	3	24	0	0	75
Lasioglossum minutissimum	Bee	0	2	0	1	0	0	3
Lasioglossum minutulum	Bee	0	1	0	0	0	0	1
Lasioglossum morio	Bee	0	6	2	2	1	0	11
Lasioglossum nitidiusculum	Bee	0	63	0	0	0	0	63
Lasioglossum parvulum	Bee	2	1	0	0	0	0	3
Lasioglossum pauxillum	Bee	2	23	4	3	3	0	35
Lasioglossum politum	Bee	0	12	0	5	0	0	17
Lasioglossum puncticolle	Bee	0	0	0	2	0	0	2
Lasioglossum quadrinotatum	Bee	0	2	0	0	0	0	2
Lasioglossum sexstrigatum	Bee	0	0	0	0	5	0	5
Lasioglossum villosulum	Bee	0	0	2	15	0	0	17
Lasioglossum xanthopus	Bee	0	0	0	2	0	0	2
Lasioglossum zonulum	Bee	0	0	0	2	3	0	5
Lasiommata maera	Butterfly	0	0	0	0	11	0	11
Lasiommata megera	Butterfly	0	0	0	0	1	10	11
Leptidea sipis/juvernica	Butterfly	0	0	0	0	13	0	13
Limenitis camilla	Butterfly	0	0	0	0	7	0	7
Lycaena dispar	Butterfly	0	0	0	0	5	5	10
Lycaena phlaeas	Butterfly	0	1	17	4	0	0	22
Lysandra bellargus	Butterfly	0	0	0	1	0	0	1
Macropis europaea	Bee	0	0	0	0	3	0	3
Maniola jurtina	Butterfly	0	29	231	22	27	31	340
Megachile centuncularis	Bee	0	0	0	0	0	1	1
Megachile circumcincta	Bee	0	0	0	2	0	0	2
Megachile lapponica	Bee	0	0	0	0	1	0	1
Megachile ligniseca	Bee	0	0	0	0	1	0	1
Megachile pilidens	Bee	0	2	0	2	0	1	5
Megachile versicolor	Bee	0	1	0	0	2	0	3
Melanargia galathea	Butterfly	0	13	42	30	0	86	171
Melanostoma mellinum	Hoverfly	20	0	17	5	6	17	65
Melanostoma scalare	Hoverfly	13	0	10	0	0	0	23
Meligramma trianguliferum	Hoverfly	0	0	1	0	0	0	1
Meliscaeva auricollis	Hoverfly	0	0	1	0	0	1	2
Meliscaeva cinctella	Hoverfly	5	0	0	0	0	0	5
Melitaea athalia	Butterfly	0	0	0	0	5	0	5
Melitaea diami	Butterfly	0	0	0	0	8	0	8
Melitaea didyma	Butterfly	0	0	0	0	0	4	4

Melitta leporina	Bee	0	1	1	3	17	0	22
Merodon analis	Hoverfly	0	0	0	0	0	1	1
Merodon equestris	Hoverfly	0	0	1	0	0	0	1
Merodon ruficornis	Hoverfly	0	0	0	0	0	1	1
Minois dryas	Butterfly	0	0	0	0	0	6	6
Myathropa florea	Hoverfly	0	1	4	1	0	5	11
Nomada flavoguttata	Bee	0	4	0	0	0	0	4
Nomada flavopicta	Bee	0	0	0	1	1	0	2
Nomada fucata	Bee	0	0	0	5	0	0	5
Nomada kohli	Bee	0	0	0	1	0	0	1
Nomiapis diversipes	Bee	0	0	0	0	0	11	11
Nymphalis xanthomelas	Butterfly	0	0	0	0	2	0	2
Ochlodes sylvanus	Butterfly	1	0	5	0	32	2	40
Osmia anthocopoides	Bee	0	3	0	0	0	0	3
Osmia aurulenta	Bee	0	0	0	0	0	1	1
Osmia bicolor	Bee	0	0	0	0	0	1	1
Osmia bicornis	Bee	0	0	0	1	0	2	3
Osmia leaiana	Bee	0	0	1	0	0	0	1
Osmia rufohirta	Bee	0	0	0	0	0	1	1
Osmia spinulosa	Bee	0	0	0	7	0	0	7
Osmia uncita	Bee	0	0	0	0	1	0	1
Panurgus calcaratus	Bee	0	71	0	11	0	0	82
Papilio machaon	Butterfly	0	0	0	0	0	2	2
Paragus bicolor	Hoverfly	0	0	0	0	0	1	1
Paragus haemorrhous	Hoverfly	2	0	0	0	0	11	13
Paragus pecchiolii	Hoverfly	1	0	0	0	0	0	1
Paragus quadrifasciatus	Hoverfly	0	0	0	0	0	1	1
Paragus testaceus	Hoverfly	0	0	0	0	0	1	1
Pararge aegeria	Butterfly	4	0	1	0	0	1	6
Pieris brassicae	Butterfly	28	6	17	8	42	44	145
Pieris napi	Butterfly	17	0	5	3	30	11	66
Pieris rapae	Butterfly	71	206	81	42	41	147	588
Platycheirus albimanus	Hoverfly	2	0	2	0	0	2	6
Platycheirus nielseni	Hoverfly	0	0	1	0	0	0	1
Platycheirus peltatus	Hoverfly	1	0	0	0	1	0	2
Platycheirus scutatus	Hoverfly	3	0	0	0	2	0	5
Platycheirus tarsalis	Hoverfly	0	0	1	0	0	0	1
Platycheirus varipes	Hoverfly	0	0	0	0	1	0	1
Plebejus argus	Butterfly	0	0	0	0	2	42	44
Plebejus argyrognomon	Butterfly	0	0	0	3	0	27	30
Polygonia c-album	Butterfly	5	0	4	0	0	0	9
Polyommatus amandus	Butterfly	0	0	0	0	18	0	18
Polyommatus bellargus	Butterfly	0	0	0	1	0	15	16
Polyommatus icarus	Butterfly	0	7	3	7	16	28	61
Polyommatus semiargus	Butterfly	0	0	0	0	8	0	8
Pontia edusa	Butterfly	0	2	0	0	0	3	5
Psilota innupta	Hoverfly	0	0	0	0	2	0	2
Pyrgus armoricanus	Butterfly	0	0	0	1	0	0	1
Pyrgus malvae	Butterfly	0	0	0	0	0	10	10
Pyronia tithonus	Butterfly	13	0	120	0	0	0	133
Rhingia campestris	Hoverfly	8	0	0	0	0	0	8
Riponnensia splendens	Hoverfly	0	0	1	0	0	0	1
Satyrium acaciae	Butterfly	0	0	0	0	0	1	1

Scaeva dignota	Hoverfly	0	0	0	0	0	45	45
Scaeva pyrastri	Hoverfly	2	0	2	1	0	3	8
Scaeva selenitica	Hoverfly	0	0	0	0	0	2	2
Seladonia kessleri	Bee	0	0	0	0	0	1	1
Seladonia subaurata	Bee	0	0	0	0	0	3	3
Seladonia tumulorum	Bee	1	0	0	0	0	0	1
Sericomyia silentis	Hoverfly	0	0	0	0	4	0	4
Speyeria aglaja	Butterfly	4	0	0	0	0	0	4
Sphaerophoria scripta	Hoverfly	232	211	64	110	155	295	1067
Sphecodes ephippius	Bee	0	0	1	1	0	0	2
Sphecodes miniatus	Bee	0	11	0	0	0	0	11
Sphecodes monilicornis	Bee	0	0	2	1	0	0	3
Sphecodes pseudofasciatus	Bee	0	0	0	1	0	0	1
Sphecodes puncticeps	Bee	0	0	0	1	0	0	1
Spilomyia diophthalma	Hoverfly	0	0	0	0	1	0	1
Syritta pipiens	Hoverfly	5	1	19	7	24	106	162
Syrphus admirandus	Hoverfly	0	0	0	0	2	0	2
Syrphus ribesii	Hoverfly	2	0	6	0	9	7	24
Syrphus vitripennis	Hoverfly	7	0	2	2	3	9	23
Systropha curvicornis	Bee	0	0	0	0	0	1	1
Systropha planidens	Bee	0	0	0	0	0	11	11
Tetralonia malvae	Bee	0	4	0	0	0	0	4
Thymelicus lineola	Butterfly	3	2	0	0	48	15	68
Thymelicus sylvestris	Butterfly	0	2	57	0	36	0	95
Vanessa atalanta	Butterfly	84	3	21	3	2	13	126
Vanessa cardui	Butterfly	0	0	0	0	1	24	25
Volucella bombylans	Hoverfly	0	0	1	1	6	0	8
Volucella inanis	Hoverfly	0	0	1	0	5	0	6
Volucella pellucens	Hoverfly	1	0	2	0	1	0	4
Volucella zonaria	Hoverfly	0	0	1	0	0	1	2
Xanthandrus comtus	Hoverfly	1	0	0	0	0	0	1
Xanthogramma dives	Hoverfly	0	0	0	0	0	3	3
Xanthogramma pedissequum	Hoverfly	0	0	0	0	1	0	1
Xylocopa violacea	Bee	0	0	0	0	0	3	3
**Total**	**All**	**1485**	**1469**	**1695**	**1102**	**1373**	**3836**	**10960**

Table S4. Generalized linear mixed model summary testing the effects of predictor variables on bee abundance in road verges

Term	Estimate	Std. Error	z value	P	
**Intercept**	2.518	0.269	9.359	< 0.001	***
**Survey Date**	-0.126	0.082	-1.550	0.121	
**Survey Date²**	-0.269	0.061	-4.441	< 0.001	***
**Nectar Abundance**	0.477	0.082	5.829	< 0.001	***
**Survey Time**	0.161	0.075	2.136	0.032	*
**Survey Time²**	-0.135	0.057	-2.362	0.018	*
**Traffic Speed**	-0.211	0.093	-2.288	0.022	*
**Daily Traffic Density**	-0.247	0.132	-1.872	0.061	
**Seasonal Traffic Density**	0.175	0.139	1.256	0.209	
**Proportion of Cropland**	0.013	0.090	0.143	0.887	
**Temperature**	-0.054	0.079	-0.685	0.493	

Table S5. Generalized linear mixed model summary testing the effects of predictor variables on bee richness in road verges

Term	Estimate	Std. Error	z value	P	
**Intercept**	1.826	0.167	10.931	< 0.001	***
**Proportion of Cropland**	-0.062	0.057	-1.088	0.277	
**Plant Species Richness**	0.190	0.077	2.487	0.013	*
**Traffic Speed**	-0.118	0.055	-2.153	0.031	*
**Latitude**	0.261	0.170	1.534	0.125	
**Seasonal Traffic Density**	-0.004	0.047	-0.090	0.928	

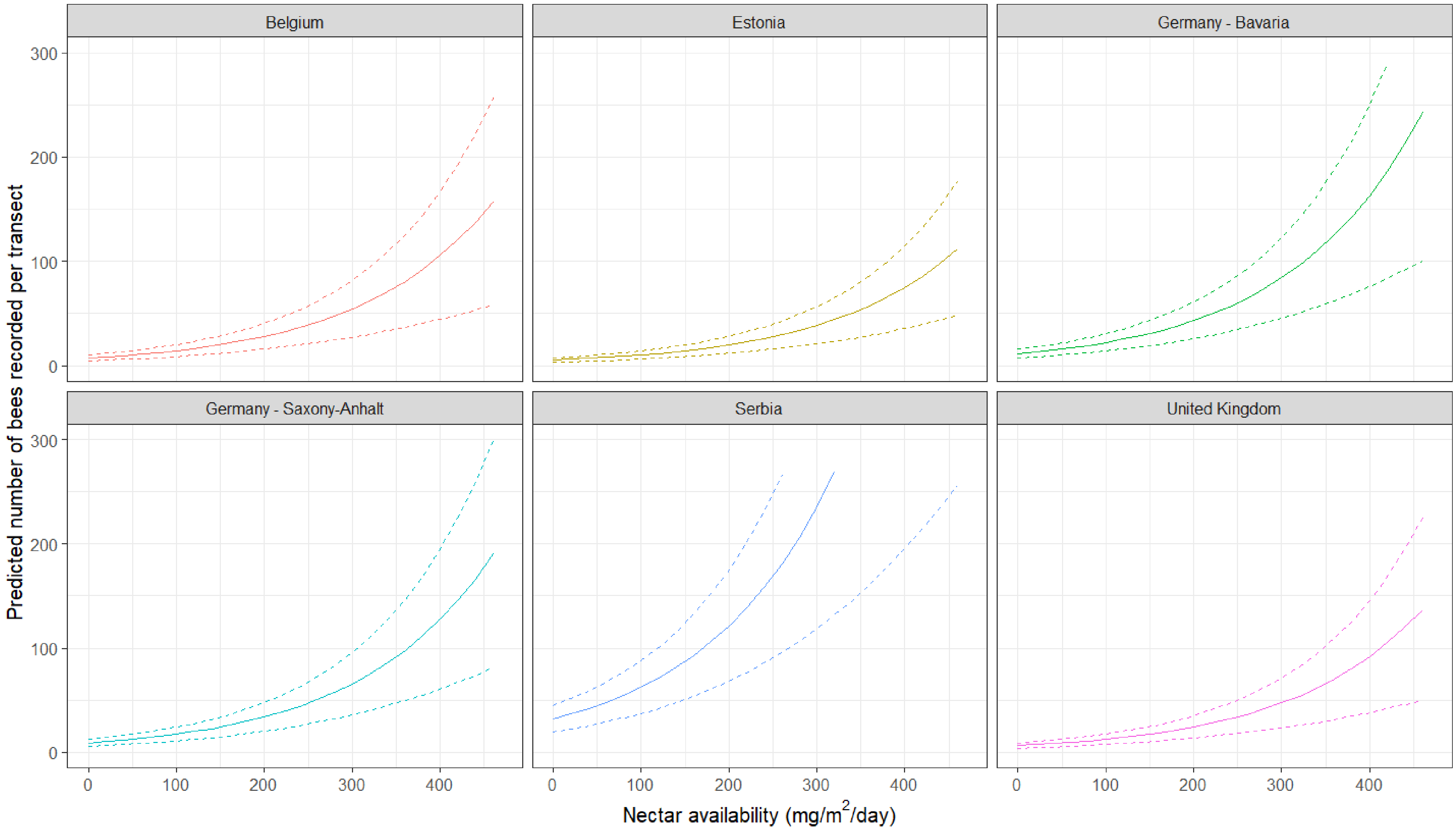

Figure S3. Predictions of bee abundance at different levels of nectar availability. All other variables were held constant at their medians.

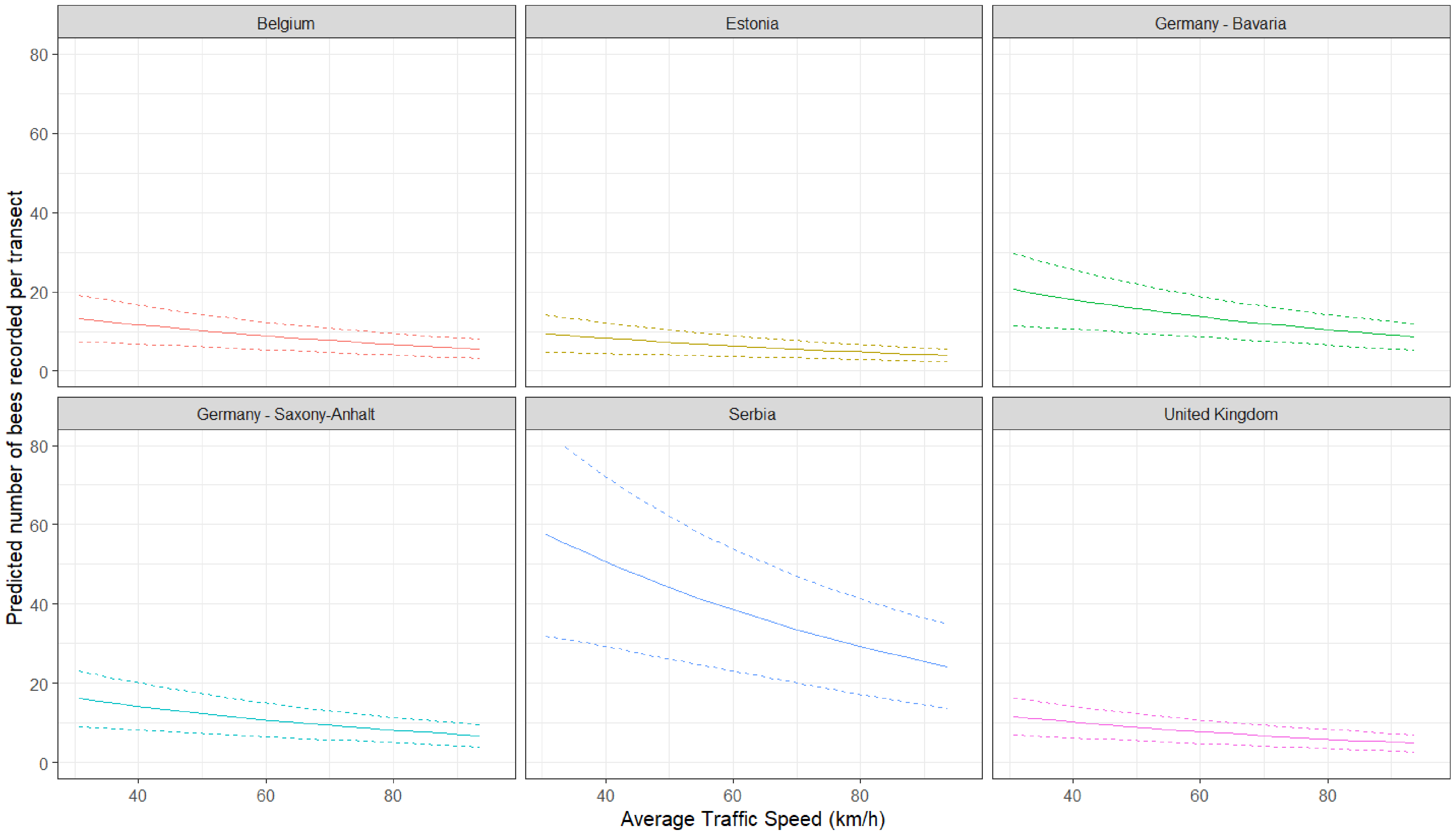

Figure S4. Predictions of bee abundance at different traffic speeds. All other variables were held constant at their medians.

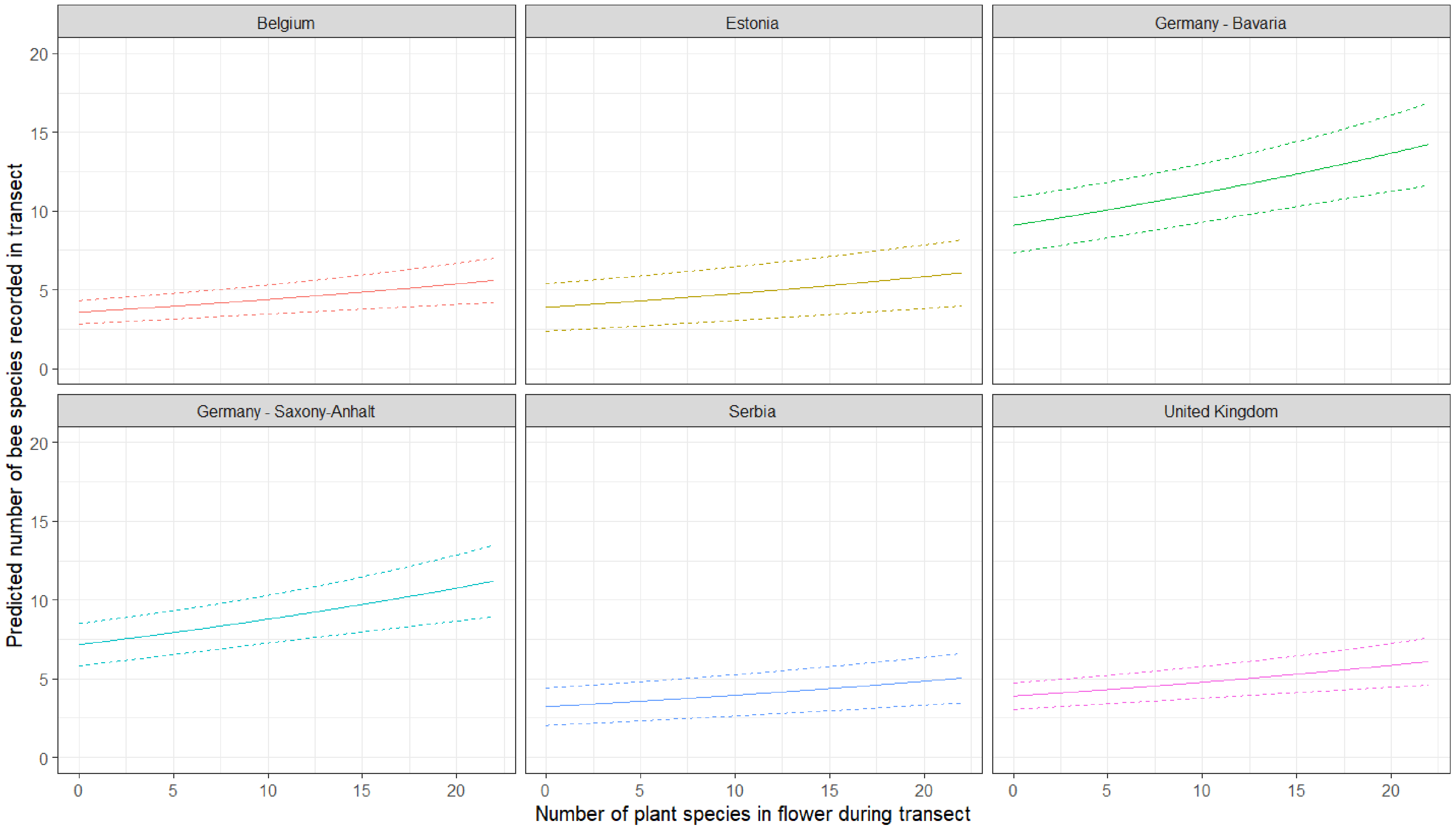

Figure S5. Predictions of bee species richness in verges containing different numbers of plant species in flower. All other variables were held constant at their medians.

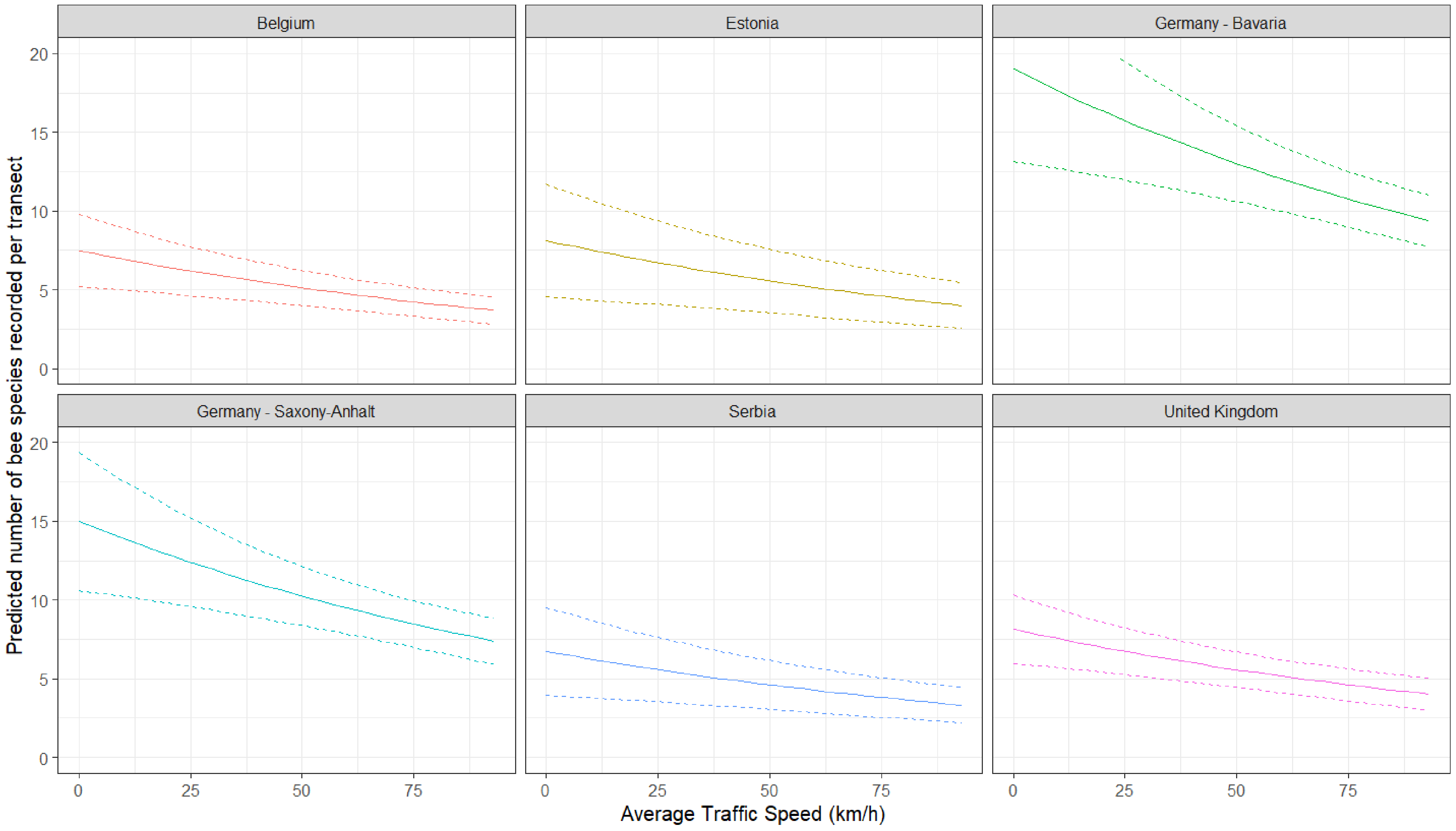

Figure S6. Predictions of bee species richness in verges alongside roads with different traffic speeds. All other variables were held constant at their medians.

Table S6. Generalized linear mixed model summary testing the effects of predictor variables on butterfly abundance in road verges

Term	Estimate	Std. Error	z value	P	
**Intercept**	1.845	0.233	7.898	< 0.001	***
**Survey Date**	0.027	0.081	0.332	0.740	
**Survey Date²**	-0.084	0.060	-1.407	0.160	
**Nectar Abundance**	0.242	0.069	3.520	< 0.001	***
**Survey Time**	0.209	0.071	2.944	0.003	**
**Survey Time²**	-0.142	0.053	-2.656	0.007	**
**Traffic Speed**	-0.171	0.086	-1.991	0.046	*
**Daily Traffic Density**	-0.058	0.130	-0.442	0.659	
**Seasonal Traffic Density**	0.100	0.137	0.072	0.943	
**Proportion of Cropland**	-0.005	0.076	-0.065	0.948	
**Temperature**	0.203	0.072	2.802	0.008	**

Table S7. Generalized linear mixed model summary testing the effects of predictor variables on butterfly richness in road verges

Term	Estimate	Std. Error	z value	P	
**Intercept**	1.641	0.090	18.312	< 0.001	***
**Proportion of Cropland**	-0.088	0.046	-1.928	0.054	
**Plant Species Richness**	0.307	0.067	4.546	0.018	***
**Traffic Speed**	0.003	0.048	0.057	0.954	
**Latitude**	0.221	0.092	2.408	0.016	*
**Seasonal Traffic Density**	-0.047	0.049	-0.973	0.330	

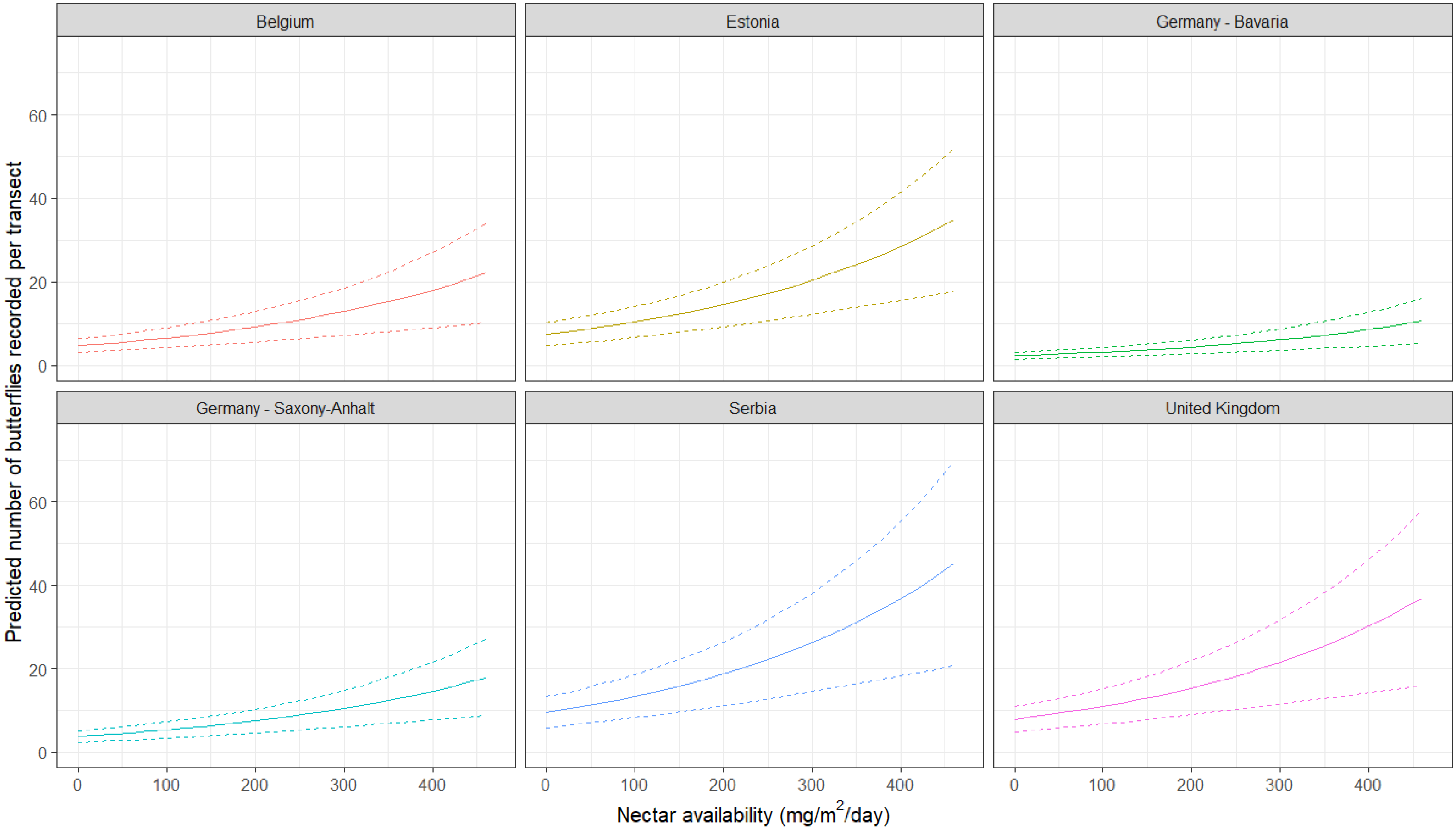

Figure S7. Predictions of butterfly abundance at different levels of nectar availability. All other variables were held constant at their medians.

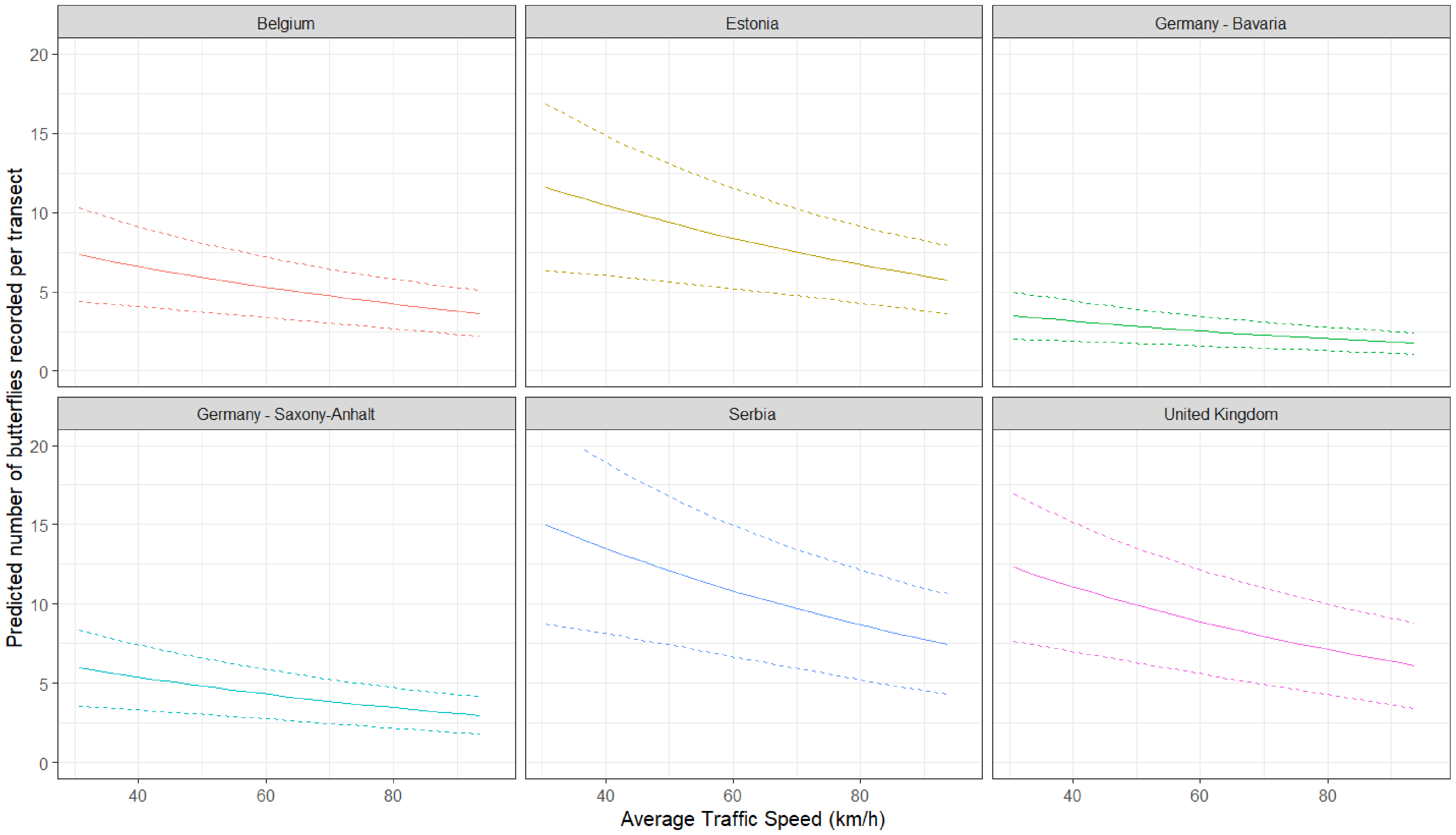

Figure S8. Predictions of butterfly abundance at different traffic speeds. All other variables were held constant at their medians.

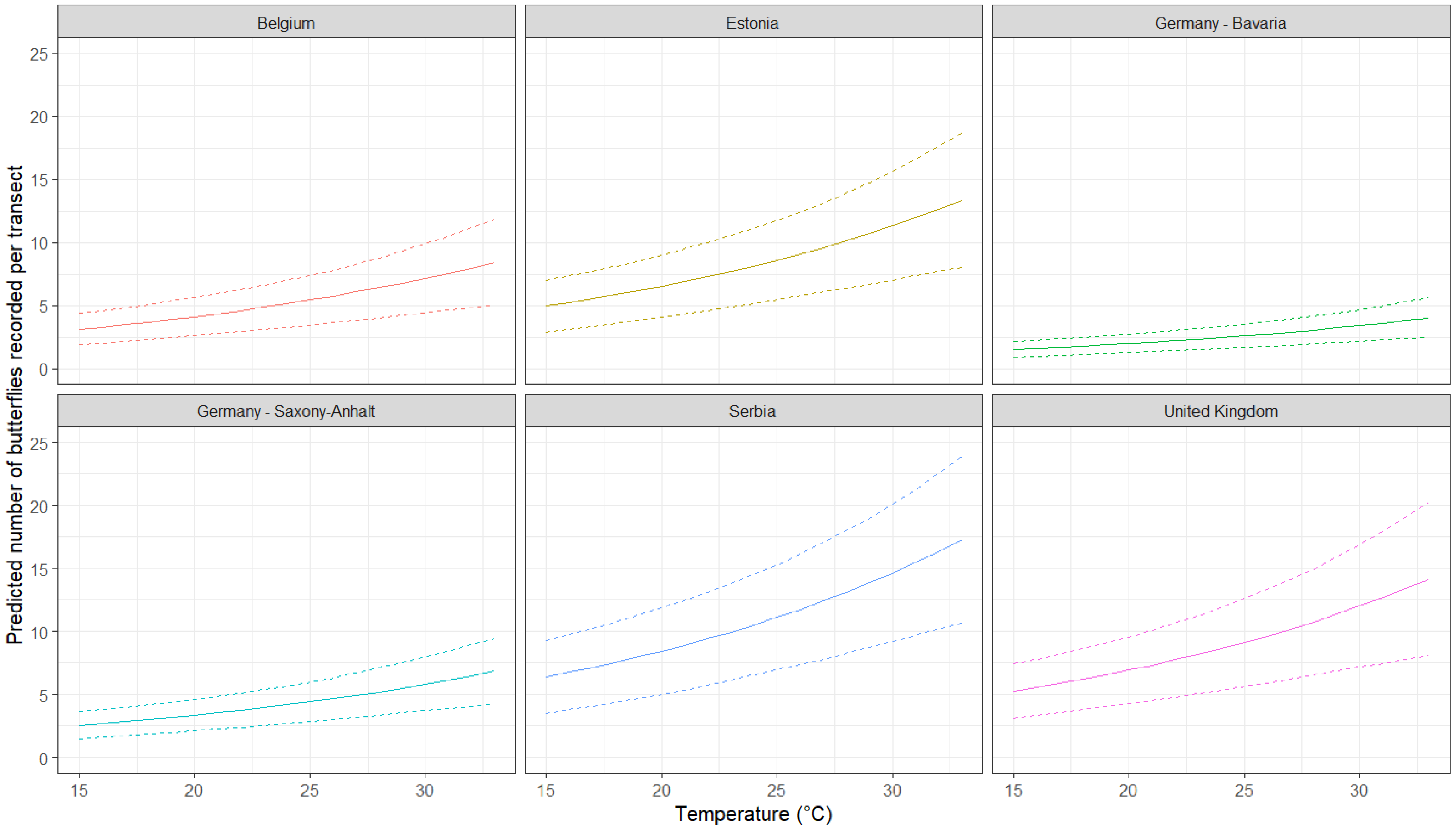

Figure S9. Predictions of butterfly abundance at different temperatures. All other variables were held constant at their medians.

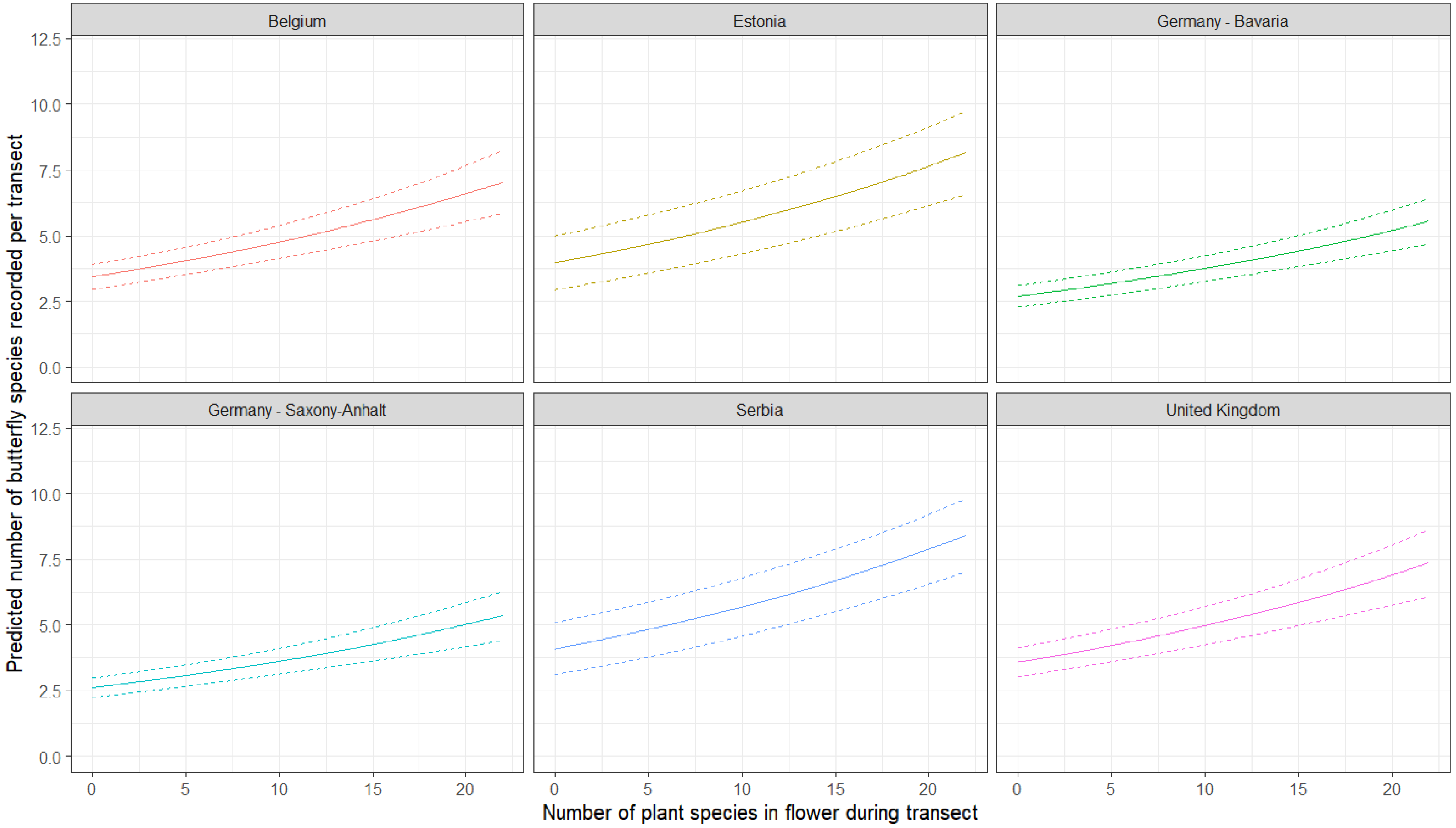

Figure S10. Predictions of butterfly species richness in verges containing different numbers of plant species in flower. All other variables were held constant at their medians.

Table S8. Generalized linear mixed model summary testing the effects of predictor variables on hoverfly abundance in road verges

Term	Estimate	Std. Error	z value	P	
**Intercept**	1.753	0.341	5.135	< 0.001	***
**Survey Date**	-0.127	0.088	-1.434	0.152	
**Survey Date²**	-0.123	0.066	-1.876	0.061	
**Nectar Abundance**	0.376	0.084	4.463	< 0.001	***
**Survey Time**	-0.235	0.085	-2.751	0.006	**
**Survey Time²**	-0.011	0.066	-0.174	0.862	
**Traffic Speed**	-0.036	0.097	-0.367	0.713	
**Daily Traffic Density**	-0.003	0.149	-0.019	0.985	
**Seasonal Traffic Density**	-0.052	0.149	-0.351	0.726	
**Proportion of Cropland**	0.052	0.089	0.586	0.558	
**Temperature**	-0.328	0.081	-4.030	< 0.001	***

Table S9. Generalized linear mixed model summary testing the effects of predictor variables on butterfly richness in road verges

Term	Estimate	Std. Error	z value	P	
**Intercept**	1.364	0.193	7.079	< 0.001	***
**Proportion of Cropland**	0.019	0.052	0.363	0.716	
**Plant Species Richness**	0.204	0.071	2.852	0.004	**
**Traffic Speed**	-0.072	0.058	-1.249	0.212	
**Latitude**	-0.110	0.196	-0.563	0.574	
**Seasonal Traffic Density**	0.053	0.050	1.066	0.289	

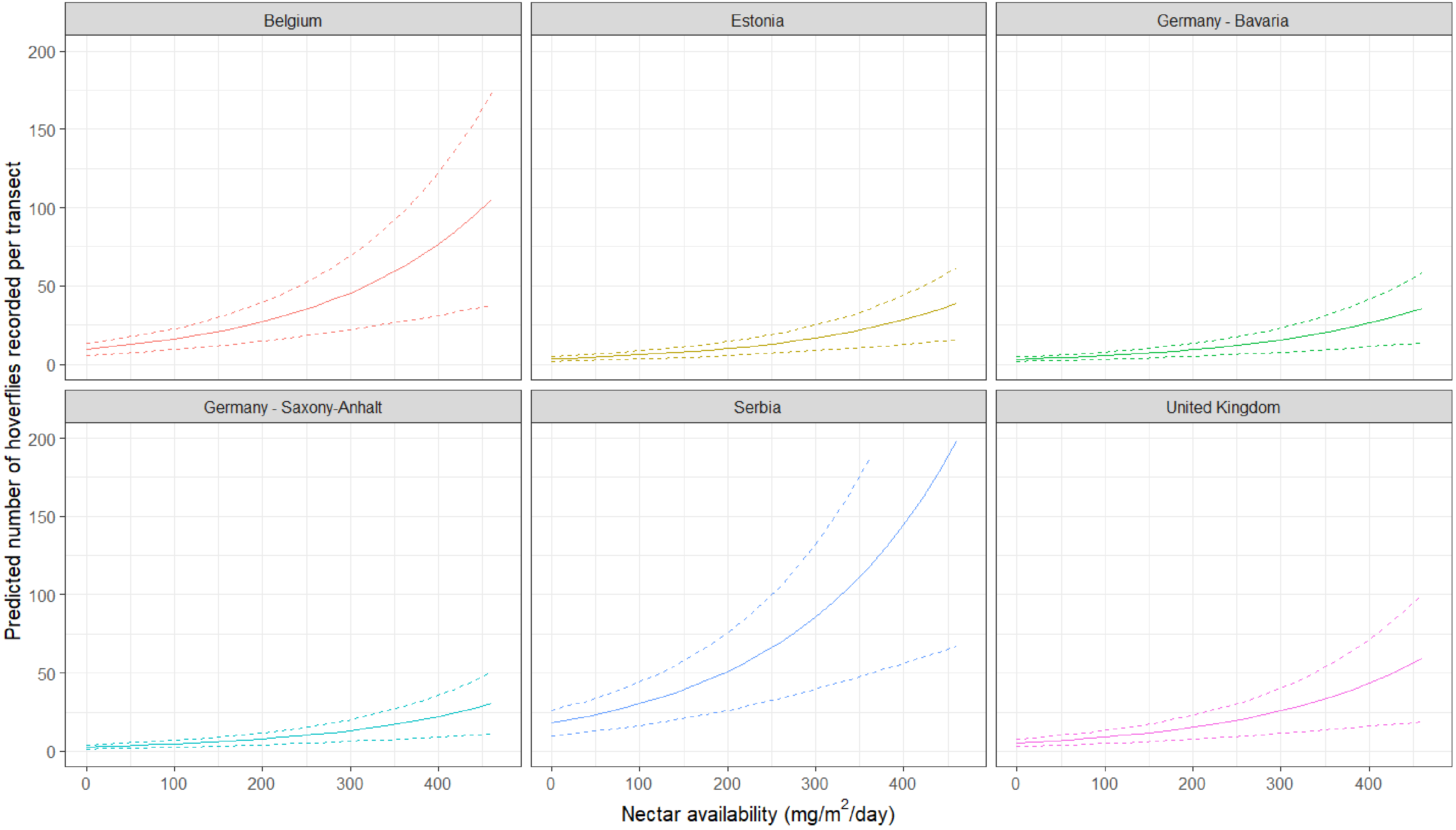

Figure S11. Predictions of hoverfly abundance at different levels of nectar availability. All other variables were held constant at their medians.

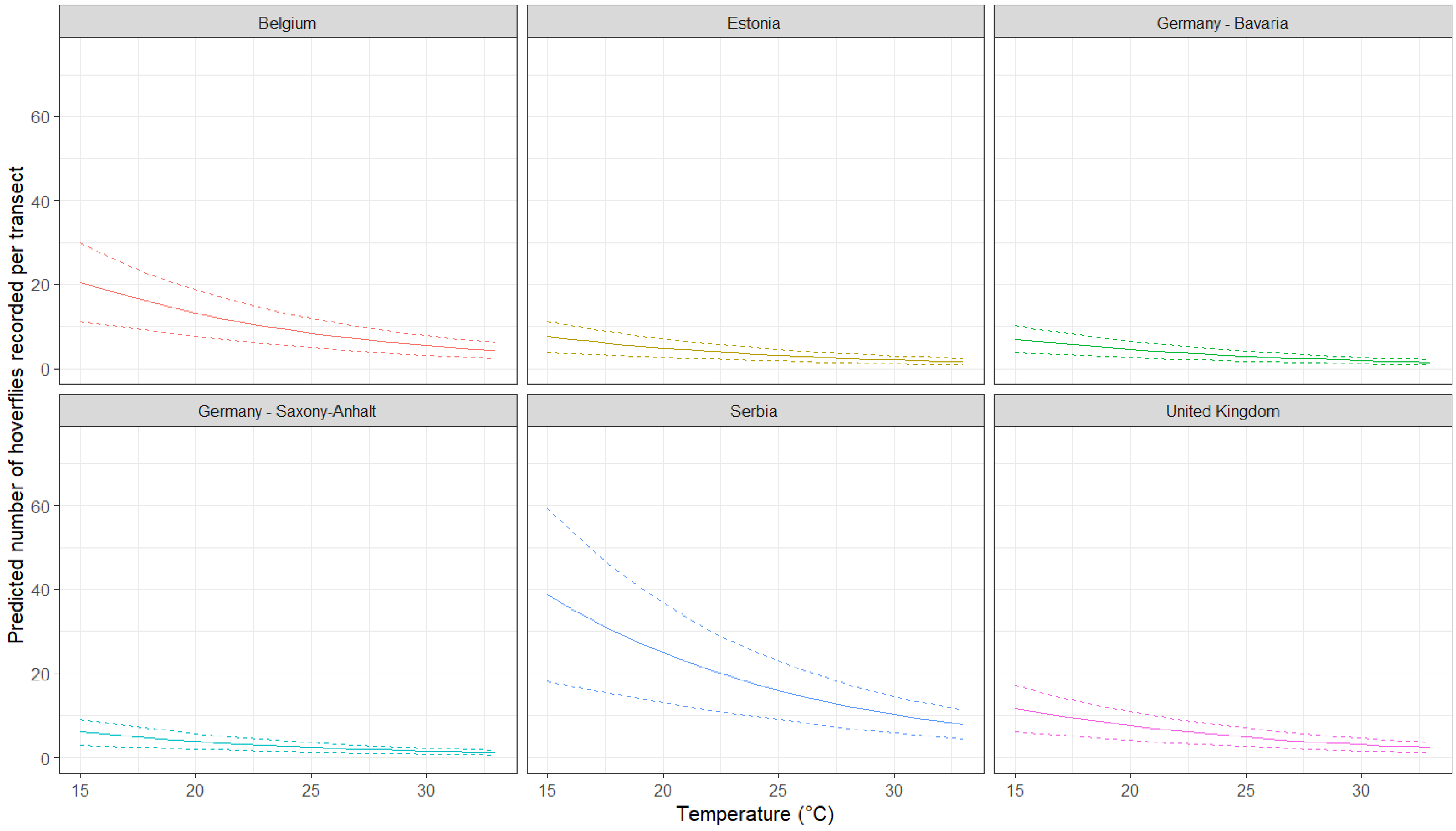

Figure S12. Predictions of hoverfly abundance at different temperatures. All other variables were held constant at their medians.

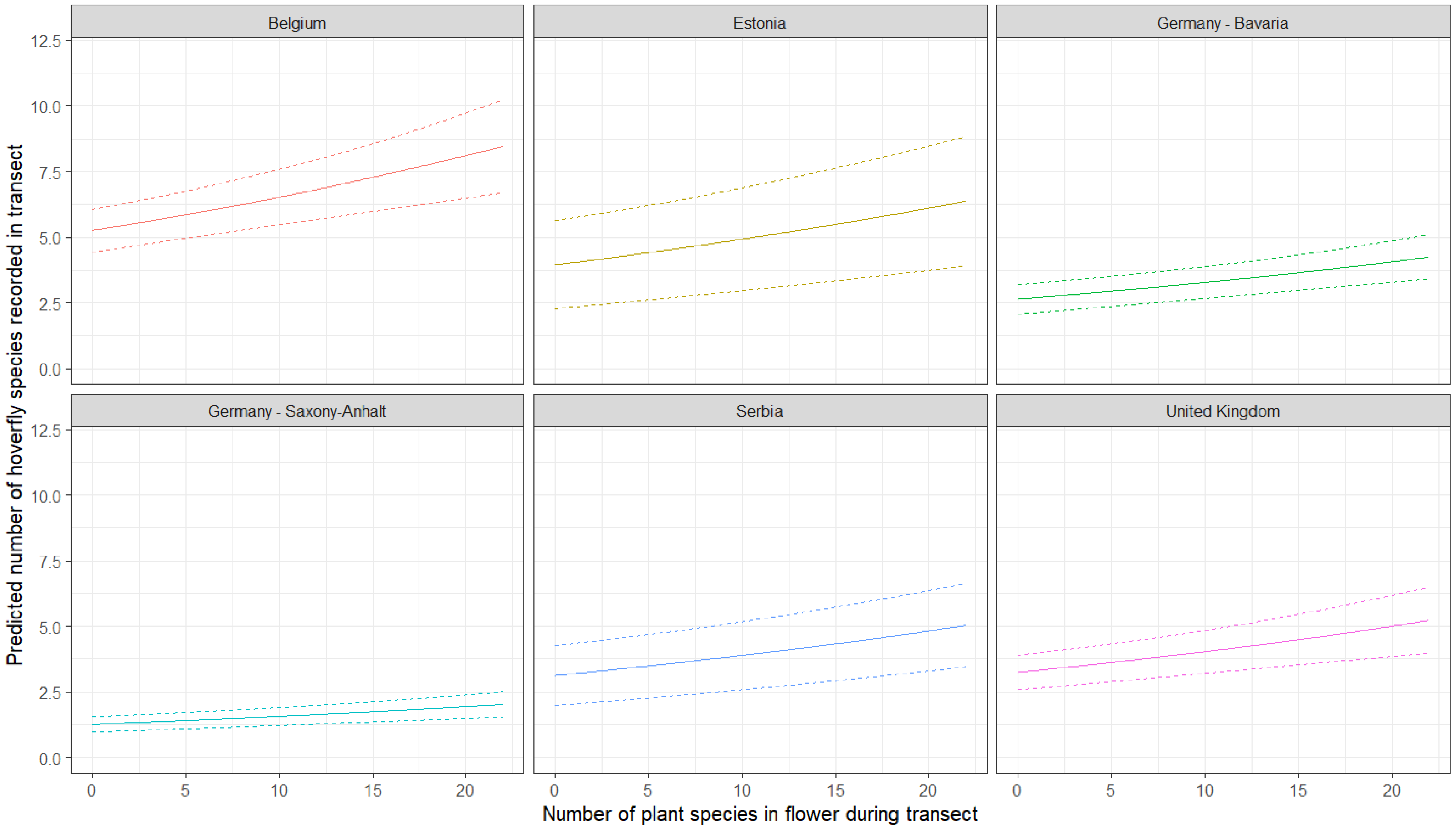

Figure 13. Predictions of hoverfly species richness in verges containing different numbers of plant species in flower. All other variables were held constant at their medians.
